# Supplementary material for: CaSpER identifies and visualizes CNV events by integrative analysis of single-cell or bulk RNA-sequencing data
Source: Nat Commun. 2020 Jan 3;11:89. doi: 10.1038/s41467-019-13779-x (PMC6941987; doi:10.1038/s41467-019-13779-x)
Supplement: Supplementary file 1 — Supplementary Information [file 41467_2019_13779_MOESM1_ESM.pdf]

# Supplementary Information

## CaSpER identifies and visualizes CNV events by integrative analysis of single-cell or bulk RNA-sequencing data

Harmanci et al.

# Supplementary Notes

## Supplementary Note 1: Accuracy of focal segments with varying segment size

We also evaluated the accuracy of focal segments at different length thresholds. To analyze the focal CNV detection performance of CaSpER, we use the segments detected by CaSpER in the TCGA-GBM bulk RNA-seq data and we computed the accuracy of CNV calls for different segment sizes. Specifically, we extracted sets of focal segments with respect to the segment length, using segments in 5 length ranges: Shorter than 1mb, between 1mb-5mb, between 5mb-10mb, between 10mb-50mb, between 50mb-100mb. We computed the segment-based PPV for the segments in these length ranges. The genotyping array data is used as ground truth while computing segment-level accuracies (Supplementary Figure 6). In general, we observed that the focal deletions and amplifications are detected with lower PPV (As low as 50% PPV) compared to the longer the segments (As high as 95% PPV).

## Supplementary Note 2: Assessing of the accessibility of CNVs by RNA-Sequencing based methods

An important factor in identifying CNVs from RNA-seq data is that the methods work at the level of genes, which means that the smallest resolution for segment coordinates will be at the gene boundaries. In other words, the regions in the genome that do not contain any genes will not be accessible for calling CNVs on them. In order to assess the accessibility of CNVs by RNA-Sequencing based methods, we randomly generated 50,000 regions in varying sizes; 100 kilobases (kB), 1 megabases (mb), 5 mb, 10 mb, 50 mb, 100 mb and computed the number of genes that overlap with them (Supplementary Fig. 7-8). Interestingly, we found that for single cell RNA-Seq datasets which cover on average 6000 genes genomewide, we found on average 0.33 genes in 100 kb segments, 2.12 genes in 1 mb segments, 10.08 genes in 5 mb segments, 20.09 genes in 10 mb segments, 102 genes in 50 mb segments, and 168 genes in 100mb. The bulk

RNA-seq datasets cover much larger portion of the genome as they measure all 20,000 protein coding genes. In bulk RNA-Seq dataset 100 Kb segments cover on average 1.15 genes, 1 mb segments cover 7.54 genes, 5 Mb segments 35.09 genes, 10 mb segments cover 68.9 genes, 50 mb segments cover 343.29 genes, and 100 mb segments cover 534.98 genes. It can be seen from these statistics that most of the segments that are shorter than 1 megabases will be very hard to detect (unless in gene dense regions) because they cover very few genes.

This has several implications: We cannot identify the CNV segments that are solely in the intergenic space (no gene overlap) and solely in the introns of genes because there is very little or no RNA-seq signal in them. Therefore, we must consider the regions in the genome without any genes in them as inaccessible for RNA-seq based CNV calling methods because the methods cannot identify CNVs there. It is worth noting that this is similar to detection of CNVs using the whole exome sequencing (WES) data except that WES can quantify the coverage on the exons so it may be able to detect breakpoints within genes and introns. But, similar to RNA-seq, WES cannot be used to detect the CNVs primarily in the intergenic space.

We assessed the performance of CaSpER using accuracy metrics at the gene level and at the segment level information (See Methods). The segment level sensitivity, however, must be carefully interpreted with above considerations in mind.

**Supplementary Note 3: Inference of CNV architecture in single-cell RNA-Seq**

For 5q:14q event pair in patient MGH31, we discovered *GFPT2* gene to be highly expressed in 5q amplified clone. The previous study has discovered that higher expression of *GFPT2* is linked with poor survival and identified *GFPT2* gene to be a potential target for therapeutic inhibition[53]. For 5q:19q event pair in patient MGH28, we discovered the *NOS2* gene to be highly expressed in 19q deletion clone. It has been previously demonstrated that *NOS2* gene is expressed in glioma stem cells and high expression of *NOS2* is correlated with decreased survival[54]. Gene expression signatures of each of the mutually exclusive clones and GO enrichment analysis are reported in

Supplementary Data 1. Since we do not have access to the cells that were analyzed and we cannot perform experimental wet-lab validation of the observed mutually exclusive patterns. However, as supportive evidence of the existence of the reported patterns and to clarify the patterns generated by CaSpER, we generated the heatmap plots of the expression signal using inferCNV as they are produced by both methods (Supplementary Fig 14). We believe that these patterns can be confirmed by visual inspection of the patterns that we are reporting can be seen in the inferCNV plot but they are not as visually discernible. The authors of the previous study do not report these results since they performed mainly a “panoramic analysis” of the results provided by inferCNV and they may not have visually identified the reported patterns. The analytical approach that CaSpER uses can systematically identify the patterns more completely compared to the panoramic analysis.

**Supplementary Note 4: Performance of CaSpER on CNVs of varying size and clonality**

We assessed the performance of CaSpER on CNVs of varying size and clonality using simulated gene expression data (See Methods). As expected, the sensitivity increases significantly with the increasing clonality (Supplementary Fig 15). We did not observe a strict relation between the sensitivity and the deletion length.

**Supplementary Note 5: Absolute copy number assignment**

An important aspect of copy number identification is the detection of genome doubling events and assignment of tumor cell ploidy (i.e. total DNA content in tumor cells). CaSpER (and many other CNV detection tools) does not assign absolute copy numbers. Assignment of absolute copy numbers and detection of genome doubling events are very challenging problems even when DNA-seq data is used with matching controls. The main reason for the hardness of absolute copy number assignment is that the RNA-seq data needs to be normalized using RPKM based normalization and after this, it is not possible to directly perform absolute quantification of copy number. If we have proper controls, such as spike-ins that are frequently generated with RNA-

seq data, the genome doubling may potentially be detected. The deletion and amplification events that CaSpER assigns are reported with respect to the baseline ploidy level of the cells (i.e., the average DNA in the genome) and these results must be correctly interpreted.

**Supplementary Note 6: Comparing read coverage and B-allele shift over the transcripts for 3'-only and full transcript sequencing**

We analyzed two different datasets to make a comparison for read coverage and B-allele shift over the transcripts. The two datasets are distinct in terms of the technology such that one is generated by Smart-Seq2 full transcript sequencing[6] and the other is generated by 10X Chromium technology[55]. Kumar et al. study generated single cell RNA-sequencing data from mouse tumor models to study cell-cell interactions. As the representative 3' sequencing data, we used the data for the sample with id "SA1N" from this study (GEO Accession number GSE121861) and mapped the reads using CellRanger software. As the representative full transcript sequencing, we used MGH31 sample's mapped reads as we have them available. For both samples, we first computed the read coverage along the genome. Next, we extracted the coverage signal over protein coding gene exons. For MGH31, we used GENCODE annotations and merged the exons for each gene, then concatenated the exons (from 5' to 3') to build the signal over each gene's exonic regions. This way, we generated the RNA-seq signal over each gene's concatenated-exons that represents the concatenation of RNA-seq signal over only the exons of each gene. We finally aggregated these exonic RNA-seq signals over all the genes. Since different genes have different total exonic lengths, we normalized the length of each gene's exonic signal to the length of the longest exonic gene in the annotation. For SA1N, we performed the same aggregation using Mouse GENCODE annotations. The aggregation of signal for MGH31 (Smart-Seq2 full transcript sequencing) and for SA1N (10X 3' sequencing) is shown in Supplementary Fig 21.

It can be seen that there is very high 3' read depth bias in 3' sequencing. For full transcript sequencing, we do observe a slight 3' bias. However, in comparison to 3' sequencing, full

transcript sequencing exhibits a much less bias when 5’ and 3’ ends are compared in terms of read depth coverage.

We next used BAFExtract to generate candidate SNVs from which we compute BAF shift. We computed the candidate SNV density over all the genes and aggregated the SNV density over every position on the genes (from 5’ to 3’). As with read depth aggregation, we normalized the length of genes to the longest gene in the annotations. These aggregations are shown in Supplementary Fig 22.

It can be seen clearly that the candidate SNVs are enriched around the 3’ end of genes for 3’ sequencing data. Full transcript sequencing data, on the other hand, shows a much more uniform coverage of the transcripts. This result indicates that the BAF shift can be reliably quantified around the ends of genes for 3’ sequencing. On the other hand, full transcript sequencing can give a more uniform and comprehensive measurement of the BAF shift signal by covering the SNVs more uniformly along with the genes with less bias on the location of SNVs.

It should also be noted that the above arguments are contingent on the fact that the suitability of CaSpER for 3’ technologies will be affected by the read depth of sequencing. Although 3’ sequencing covers the 5’ ends of genes to a much lesser extent than 3’ ends, there is still some coverage on the 5’ ends of sequences as seen in above plots. If both technologies are used with decent coverage, the read coverage over the SNVs (even around 5’ ends of genes) can provide similar power to measure BAF shift. Consequently, both technologies can provide useful BAF information with decent sequencing depths.

**Supplementary Note 7: Applying CaSpER on 10x single cell RNA-Seq data**

We assessed the suitability of CaSpER 3’ transcript sequencing data using the multiple myeloma 10X RNA-Seq data (MM135) presented on HONEYBADGER study[21]. Even though the exact

ground truth CNV calls are unknown for this study, we show the concordance of CaSpER CNV calls with HONEYBADGER.

This MM135 data contains normal and MM cells, therefore, we first performed TSNE to separate MM and normal cells. We also checked expression levels of MM markers (SDC1, CD38) in all cells to better identify MM cells. We next normalized the expression values of MM cells using normal cells. We plotted heatmap of median filtered expression data (Supplementary Fig. 23). We observed large scale deletion events in chromosome 13, 18 and 22 from gene expression heatmap. BAF shift plot showed concordant shifts in chromosome 13 and 18 (Supplementary Fig 24). We also calculated large scale event summary for MM135 (Supplementary Fig 25). As it is seen from the below large scale CNV event summary plot, CaSpER identified various large scale CNV events including chromosome 13 and 22 as expected (Supplementary Fig 25) Moreover these events are also reported in HONEYBADGER paper. In summary, we recommend using CaSpER on 10x data. However, data preprocessing steps should be performed carefully because 10x has different biases compared to SMART-Seq data. We added documentation specifically for 10x RNA-Seq data at our website.

**Supplementary Note 8: The performance difference in the bulk and single cell RNA-seq data.**

Several technical and biological factors are underlying the higher sensitivity of CNV detection for bulk RNA-Seq data. We discuss these factors below.

The first factor is that DR-Seq technology or other simultaneous RNA-DNA sequencing techniques are not standardized, unlike the genotyping arrays where we can directly identify known CNV calls. This would mean that the technical artifacts in the DR-Seq technology are potentially affecting the accuracy of the results.

The second factor is that, in DR-Seq study, absolute copy numbers are assigned to the segments using a computational approach. However, both CaSpER and HoneyBADGER assigns relative copy numbers to the detected segments where copy numbers are assigned with respect to the average DNA content in each cell. Therefore, we processed the absolute copy numbers from DR-Seq study to build the relative copy numbers (i.e., amplification, deletion, neutral) in each cell. The difference in calculating relative copy number calls among different datasets might lead to poor performance.

The third factor is that, as we have stated earlier, the CNV calling methods work at the level of genes, which means that the smallest resolution for segment coordinates will be at the gene boundaries. In other words, the regions in the genome that do not contain any genes will not be accessible for calling CNVs on them. Single cell RNA-Seq datasets cover on average 6000 genes genomewide, whereas bulk RNA-Seq dataset measure all 20,000 protein-coding genes. Most of the segments that are shorter than 1 megabases will be very hard to detect (unless in gene dense regions) because they cover very few genes. We believe that these should be kept in mind while interpreting the accuracy results. This has several implications: We cannot identify the CNV segments that are solely in the intergenic space (no gene overlap) and solely in the introns of genes because there is very little or no RNA-seq signal in them. This impacts the way that the accuracy is estimated because inclusion of inaccessible regions in sensitivity estimation will adversely (and unfairly) decrease the sensitivity of the methods.

The fourth factor is that DR-Seq data is applied to metastatic breast cancer cell line (SK-BR-3) harboring very complicated diverse chromosomal alterations. Following our previous point, the complex CNV profiles of SK-BR-3 cell line is affecting sensitivity. It should, however, be noted that even for the complex ovarian cancer the accuracy of the CNV detection of bulk RNA-seq is around 60% TPR. Therefore, other factors are affecting probably more.

The fifth factor is the limited number of cells. CaSpER will have better estimates with more cells.

Each of the above mentioned factors impacts the discordancy between single cell and bulk RNA-seq CNV detection accuracies. These factors are generally fairly hard to control since matching both the technical and biological covariates require extremely well-characterized samples and very strict sample preparation and sequencing checks.

**Supplementary Note 9: Impact of point-mutations, RNA-editing, amplification errors, and sequencing in CNV identification from RNA-Seq**

While amplification errors and sequencing errors impact other methods such as DNA-seq based detection, RNA-editing impacts specifically RNA-seq based detection of CNVs and it introduces potentially adverse biases.

Point mutations impacting B-allele frequency. The statement becomes circular when we use the B-allele frequency of SNVs to identify CNVs and claim that the detecting CNVs before detecting SNVs is useful for correct identification of SNVs. We must, therefore, clarify this. While we agree that the individual point mutations affect the B-allele frequency, we are not using the B-allele frequency of individual variants; we are using a collective shift in the observed B-allele frequencies of consecutive variants. This is the main reason why we use the multiscale smoothing B-allele shift signals while assigning the CNV calls. The smoothing summarizes the estimated B-allele shift signal among potential consecutive SNVs. We are therefore claiming that one can detect the B-allele shift without the existence of a high-quality set of SNVs. We believe that the concordance between our B-allele shift plots (Generated without a high quality SNV call set) and the expression signals is convincing evidence that supports this claim.

RNA-editing is a general mechanism where RNA is edited post-transcriptionally by cells. The nucleotide sequence of the transcripts is changed as a result. As the RNA sequence is changed, the sequenced RNA reads will reflect the nucleotide modifications. While these edited nucleotides may not affect the gene expression quantifications (Assuming they do not interfere with read

mapping), they may potentially impact the BAF shift information because the modified nucleotides may look like SNVs while reads are being processed. The RNA editing events are mainly dominated by A-to-I modifications. While the exact number of RNA editing sites are not known, the common RNA editing events are hypothesized to be rare, on the order of 10,000-100,000 sites[56,57] and many of the detected editing events overlap with repeat regions and do not overlap with coding sequences.

Since the number of the events is low, we believe that these events will not have a major impact on the BAF shift generation. Secondly, the RNA-seq data is predominantly enriched in the coding and exonic sequences. Since there is a high association of RNA-editing events with repetitive non-coding elements, we believe that they will have an almost unnoticeable impact on the BAF shift signal measured on the exonic regions. However, the cancer cells may still have high RNA editing just because the editing pathways may be malfunctioning. Therefore, we believe the impact of RNA editing should be considered carefully any time RNA-Seq data is used to identify CNVs.

Amplification errors stem from the PCR amplifications that are used to increase the cDNA content in the sequencing libraries. PCR amplification may introduce errors that get amplified within any cycle. Most alarmingly, these errors are correlated among different reads and it is hard to correct for these errors. These amplified errors may seem like novel mutations. Especially in the lack of a matching control data, these PCR errors may adversely impact BAF shift signal generation. We hope that the other less biased PCR-free amplification assays will be more available to decrease the amplification errors. In addition, molecular barcoding such as UMIs may be useful to detect duplicates and remove them effectively.

Sequencing errors are introduced in the sequencing step when the sequencing machine wrongly assigns a base. These errors are less of a concern then PCR amplifications since they are generally independently introduced in different reads but this may not always be the case because

sequencing errors can be context specific. Nevertheless, the sequencing error rates of short read sequencing technologies such as Illumina is very low; around 0.1% per base[58]. We therefore think the sequencing errors will impact BAF shift generation slightly.

## Supplementary Note 10: Accuracy of BAF shifts signal estimated by CaSpER

We expect that regions with amplification or deletion to be mostly accompanied with loss of heterozygosity (LOH) and harbor BAF shift except for homozygous deletions and balanced amplifications. In most of the cases, we do not have matched normal RNA-Seq samples, therefore; we predict the BAF shift signal only from tumor RNA-Seq samples. To evaluate the accuracy of the BAF shift signal from CaSpER, we compared our BAF shift signal with the signal generated from GATK best practices workflow for RNA-Seq variant calling. For this, we first used GATK to call SNVs using the tumor RNA-seq data. Next, we used the heterozygous calls from the tumor and computed the GATK based BAF shift signal. We did not observe any particular features in the GATK-based BAF shift signal that will provide extra benefit for CNV detection compared to CaSpER's BAF shift signal (Supplementary Fig 26). It is important to note that CaSpER performs several SNV filtering steps to remove variants that may bias BAF shift signal adversely (Methods Section). This result supports our hypothesis that BAF shift signal detection for CNV identification can be performed without reliance on a good variant call set.

## Supplementary Note 11: Change of HMM model parameters with increasing number of cells

The exact ground truth CNV calls are unknown for both MM135 and MGH31 study. However, based on CaSpER and HONEYBADGER calls we know that most of the MM cells in MM135 harbor large scale chromosome 13 and 18 deletions. Therefore we first randomly selected n=7, n=50 and n=100 cells among the MM135 cells that harbor 18p and 13q deletion. When we sampled n=7 cells, the accuracy of calling chromosome 13 and 18 deletions were 71%, whereas with n=50 accuracy was 92% and with n=100 cells accuracy was 94%. Supplementary Fig. 33

shows the large scale CNV event calls for chromosome 18 and 13 for sampled n=7, n=50 and n=100 cells. As it is seen from Supplementary Fig. 33, concordance of CNV calls increases with the number of cells. We calculated the mean values of the normal distributions corresponding to 5 copy number states (q1:homozygous deletion, q2:heterozygous deletion, q3:neutral, q4:amplification, q5:high-level amplification) that are derived from randomly selected n=7, n=50, n=100 cells and all cells in MM135 dataset. We observed that the initial HMM model parameters change when different numbers of cells are analyzed (Supplementary Fig. 34).

Similarly in MGH31, based on CaSpER and HONEYBADGER calls we know that most of the MGH31 cells harbor chromosome 10 deletion. Therefore we first randomly selected n=7 and n=50 cells among the MGH31 cells that harbor 10p and 10q deletion. When we sampled n=7 cells accuracy of calling chromosome 10 deletions were 35%, whereas with n=50 accuracy was 99%. Supplementary Fig. 35 shows the large scale CNV event calls for chromosome 18 and 13 for randomly selected n=7 and n=50 cells. As it is seen from Supplementary Fig. 35, the concordance of CNV calls increases with the number of cells. We calculated the mean values of the normal distributions corresponding to 5 copy number states (q1:homozygous deletion, q2:heterozygous deletion, q3:neutral, q4:amplification, q5:high-level amplification) that are derived from randomly selected n=7, n=50, n=100 cells and all cells in MM135 dataset. We observed that the initial HMM model parameters change when different numbers of cells are analyzed (Supplementary Fig. 36).

**Supplementary Note 11: Extension of CaSpER to other functional genomics datasets**

In this paper, we have focused on the detection of CNV events from RNA-Seq datasets. This is because RNA-sequencing is becoming an everyday tool in research and clinical settings. Also, the number of RNA-sequencing datasets is increasing comparably with the whole genome and whole exome sequencing datasets. Although we have focused only on RNA-sequencing data, the analysis framework that CaSpER utilizes can be extended to other functional genomics datasets such as ChIP-Sequencing, which are currently not performed as often as RNA-

sequencing. We hypothesize that the CNV architecture can be reliably detected using these functional genomics datasets jointly. As more cancer epigenomics datasets are generated, CaSpER can be tuned to analyze data from these assays for detection of copy number and LOH events.

**Supplementary Note 12: Experimental design optimization using CaSpER**

Even though RNA-seq and WGS are performed regularly on the same samples, we foresee that experimental designs can first start by performing RNA-seq rather than starting with WCGS. This will enable the researchers to study the samples simultaneously from the genetic and the transcriptomic perspective. After this evaluation, the researchers can focus on the uncharacterized samples, for example, samples that do not harbor any of the known large scale CNVs associated with known subtypes of cancer. After selecting the uncharacterized samples, the researchers can continue with performing WGS of these uncharacterized samples. This “sequential” or “RNA-seq first” design is advantageous for several reasons. First, the cost can be decreased significantly. WGS is currently much more expensive in terms of sequencing, analysis, and storage compared to the costs associated with RNA-seq. If the researchers can decrease the number of samples for which WGS is performed by focusing on the most interesting samples, this can decrease the costs substantially. Additionally, RNA-seq can be used to identify much more information such as the transcript fusion events that may have important clinical implications. While we are optimistic with the RNA-seq first design, we are just presenting this case as a potential example and we do not want to overstate this scenario because the sample preselection may create adverse statistical biases that must be properly controlled.

## Supplementary References


53 Panosyan EH, Lin HJ, Koster J, Lasky JL. In search of druggable targets for GBM amino acid metabolism. *BMC Cancer* 2017;**17**:. https://doi.org/10.1186/s12885-017-3148-1.

54 Eyler CE, Wu Q, Yan K, MacSwords JM, Chandler-Militello D, Misuraca KL, *et al.* Glioma stem cell proliferation and tumor growth are promoted by nitric oxide synthase-2. *Cell* 2011;**146**:53–66. https://doi.org/10.1016/j.cell.2011.06.006.

55 Kumar MP, Du J, Lagoudas G, Jiao Y, Sawyer A, Drummond DC, *et al.* Analysis of Single-Cell RNA-Seq Identifies Cell-Cell Communication Associated with Tumor Characteristics. *Cell Rep* 2018;**25**:1458–1468.e4. https://doi.org/10.1016/j.celrep.2018.10.047.

56 Porath HT, Knisbacher BA, Eisenberg E, Levanon EY. Massive A-to-I RNA editing is common across the Metazoa and correlates with dsRNA abundance. *Genome Biol* 2017;**18**:185. https://doi.org/10.1186/s13059-017-1315-y.

57 Bahn JH, Lee J-H, Li G, Greer C, Peng G, Xiao X. Accurate identification of A-to-I RNA editing in human by transcriptome sequencing. *Genome Res* 2012;**22**:142–50. https://doi.org/10.1101/gr.124107.111.

58 Glenn TC. Field guide to next-generation DNA sequencers. *Mol Ecol Resour* 2011;**11**:759–69. https://doi.org/10.1111/j.1755-0998.2011.03024.x.

## Supplementary Figures

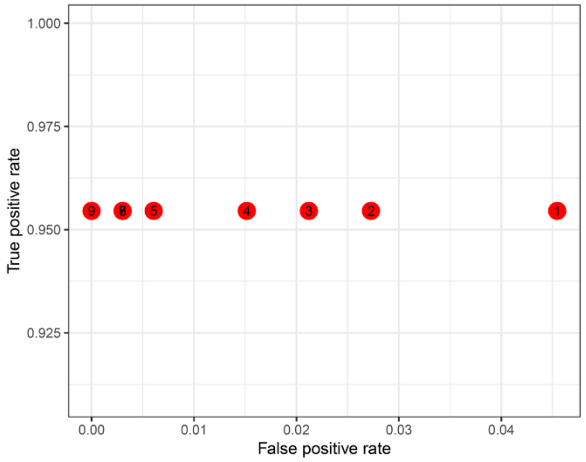


**Supplementary Figure 1. TPR and FPR values for bulk Meningioma deletion CNV events with varying $\gamma$ thresholds.** Plot shows the large scale event performance which is assessed using genotyping array. Labels in red points represent the $\gamma$ threshold.

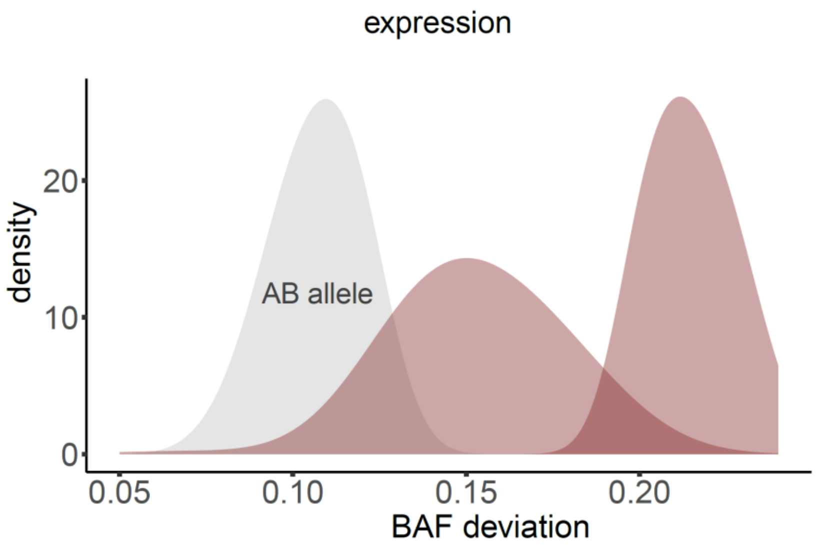


**Supplementary Figure 2. BAF shift threshold is estimation.** BAF shift threshold is estimated by fitting Gaussian mixture model (GMM). GMM identified three classes of BAF shift groups where the first group corresponds to no shift regions whereas the second and the third group corresponds to BAF shift regions with loss or amplification events.

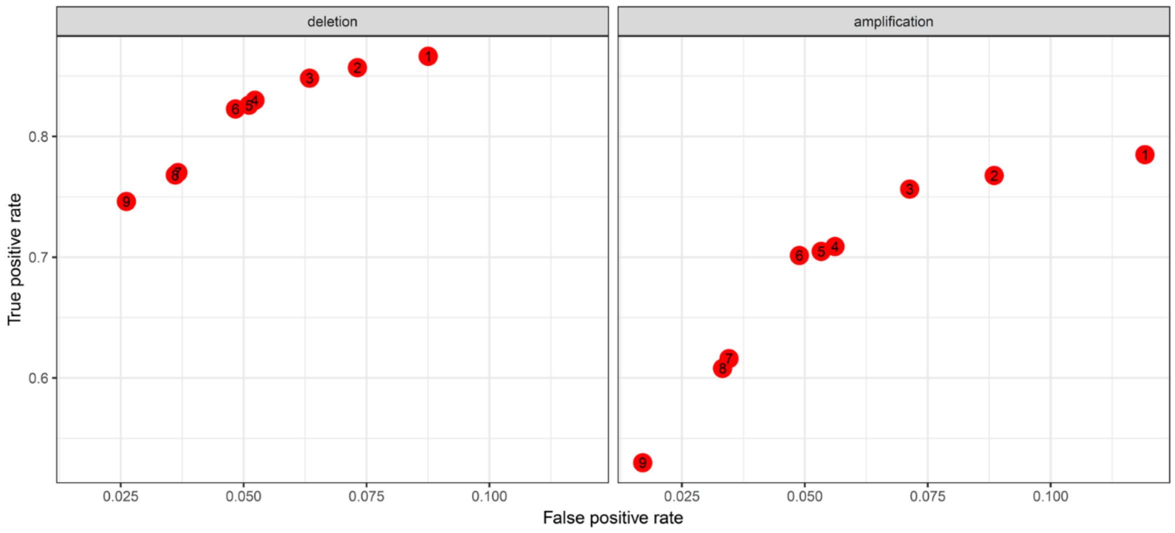


**Supplementary Figure 3.** TPR and FPR values for bulk TCGA-GBM RNA-Seq data with varying $\gamma$ thresholds. Plot shows the gene based performance which is assessed using genotyping array. Labels in red points represent the $\gamma$ threshold.

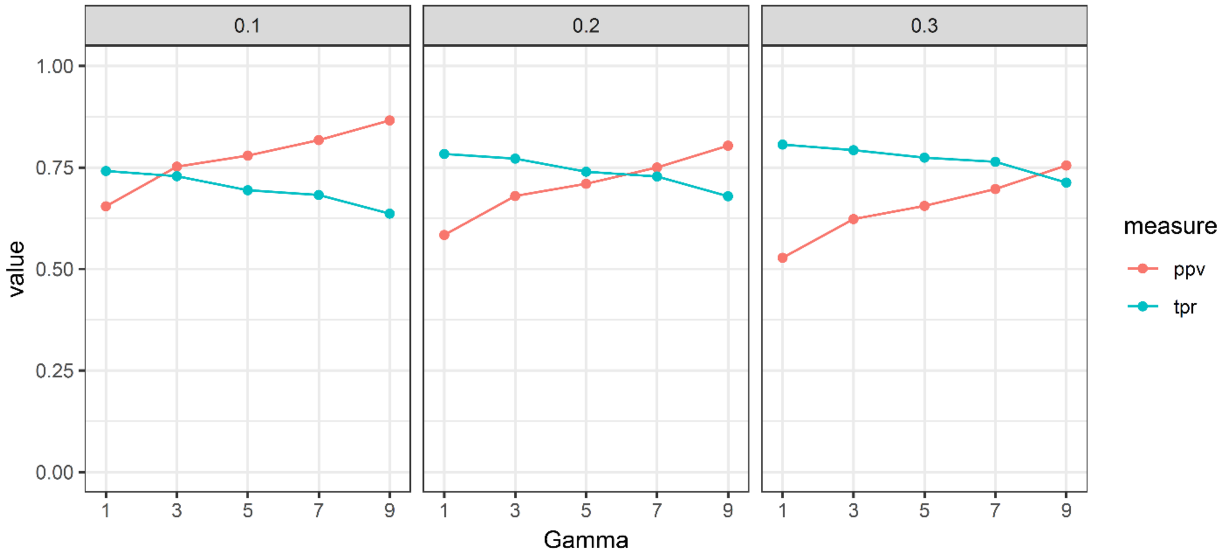


**Supplementary Figure 4.** TPR and PPV values for deletion events estimated from bulk TCGA-GBM with varying $\gamma$ thresholds and varying segment mean thresholds for genotyping array (0.1, 0.2 and 0.3). Plot shows segment based performance which is assessed using genotyping array.

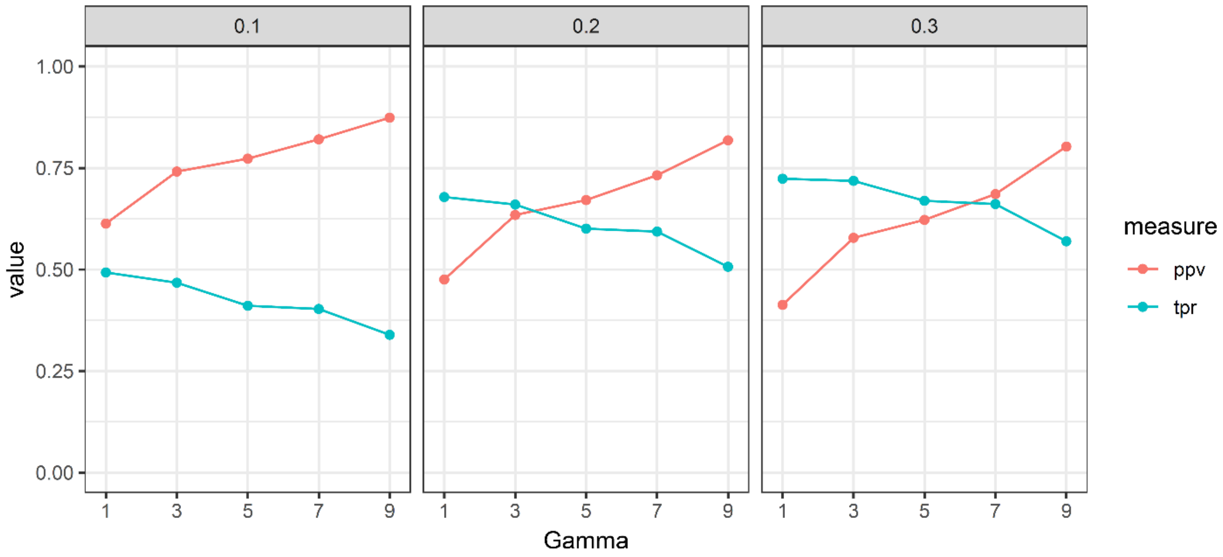


**Supplementary Figure 5.** TPR and PPV values for amplification events estimated from bulk TCGA-GBM with varying $\gamma$ thresholds and varying segment mean thresholds for genotyping array (0.1, 0.2 and 0.3). Plot shows segment based performance which is assessed using genotyping array.

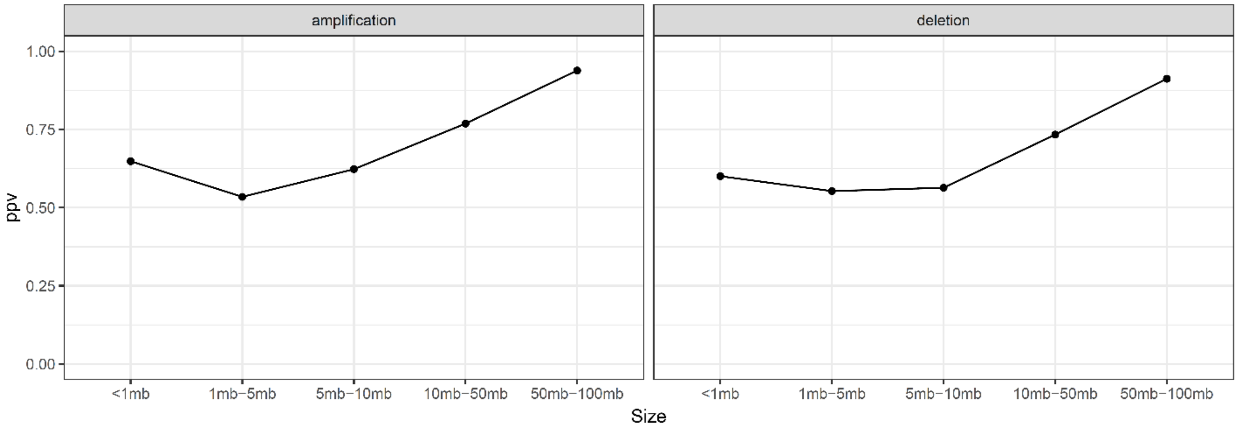


**Supplementary Figure 6. Segment based accuracy measures for bulk TCGA-GBM with varying segment size intervals (<1mb, 1mb-5mb, 5mb-10mb, 10mb-50mb, 50mb-100mb).** Performance is assessed using genotyping array.

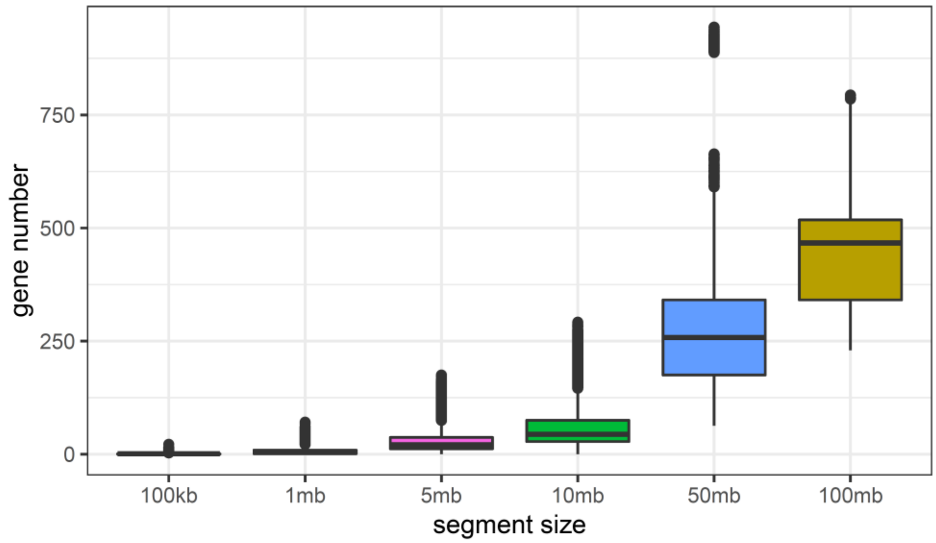


**Supplementary Figure 7. Accessibility of CNVs by bulk RNA-Sequencing based methods.** X-axis corresponds to regions in varying sizes; 100 kilobases (kB), 1 megabases (mb), 5 mb, 10 mb, 50 mb, 100 mb and Y-axis corresponds to the number of genes that overlap with these 50000 simulated regions with varying sizes. Lines depict the median values; boxes plot 25th to 75th percentiles, whereas separately plotted dots show the outliers.

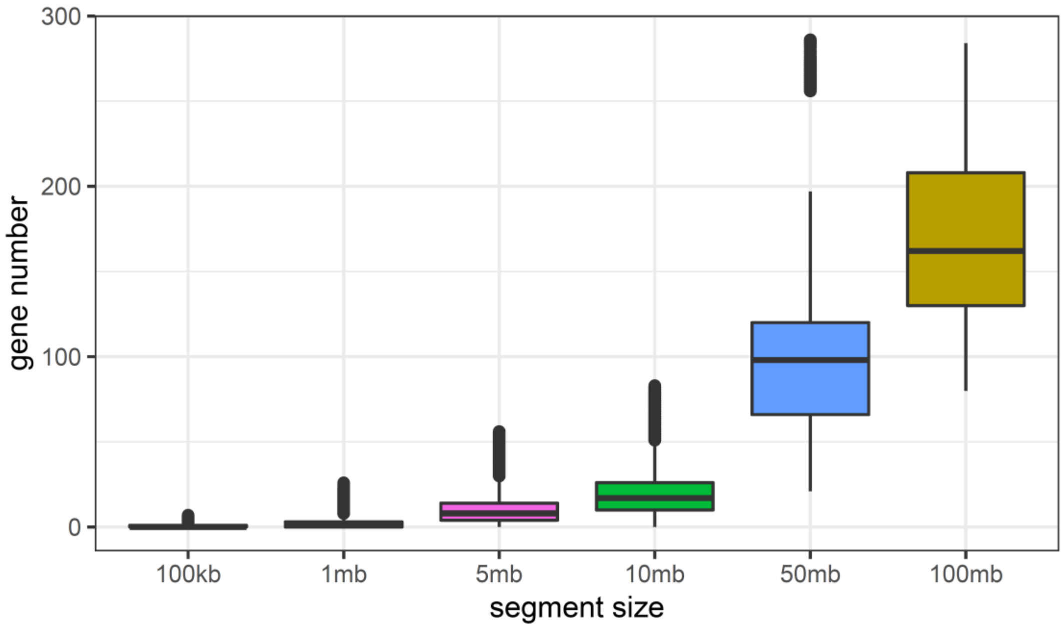


**Supplementary Figure 8. Accessibility of CNVs by single-cell RNA-Sequencing based methods.** X-axis corresponds to regions in varying sizes; 100 kilobases (kB), 1 megabases (mb), 5 mb, 10 mb, 50 mb, 100 mb and Y-axis corresponds to the number of genes that overlap with these 50000 simulated regions with varying sizes. Lines depict the median values; boxes plot 25th to 75th percentiles, whereas separately plotted dots show the outliers.

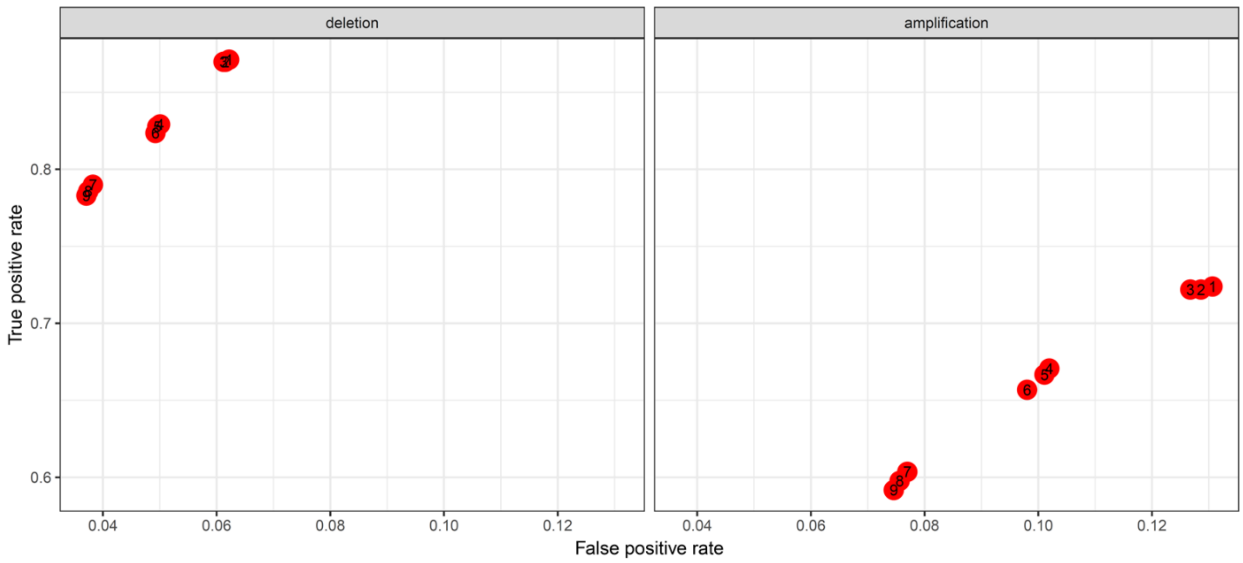


**Supplementary Figure 9. TPR and FPR values for bulk TCGA-BRCA RNA-Seq data with varying $\gamma$ thresholds.** Plot shows the large scale event performance which is assessed using genotyping array. Labels in red points represent the $\gamma$ threshold.

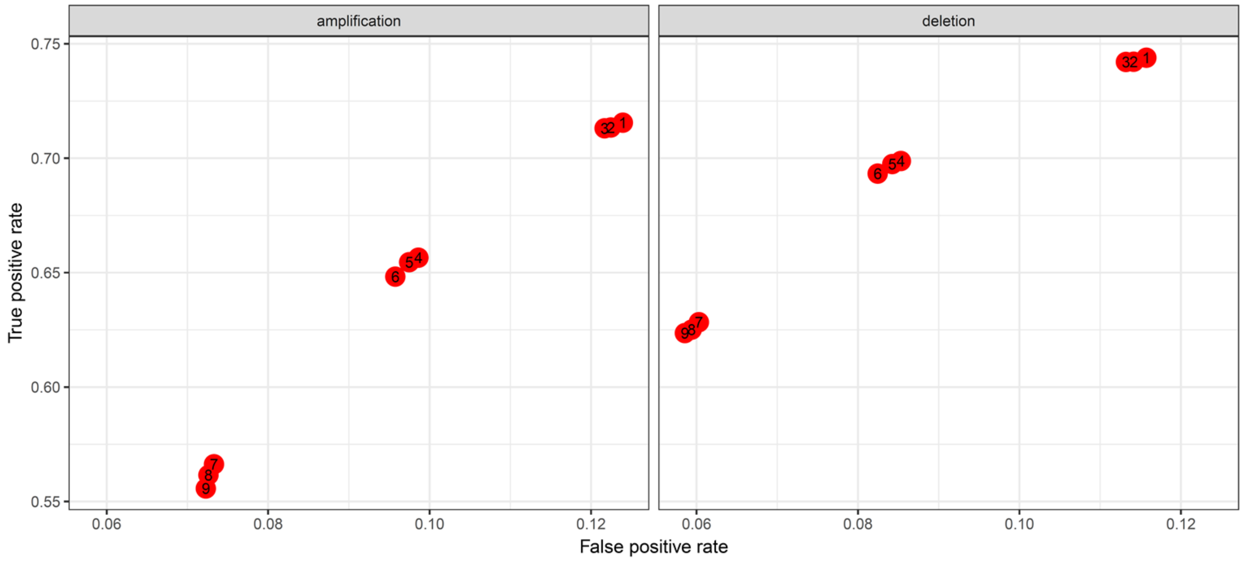


**Supplementary Figure 10. Gene based performance on TCGA-BRCA data.** Plot shows the gene based performance which is assessed using genotyping array. Labels in red points represent the $\gamma$ threshold.

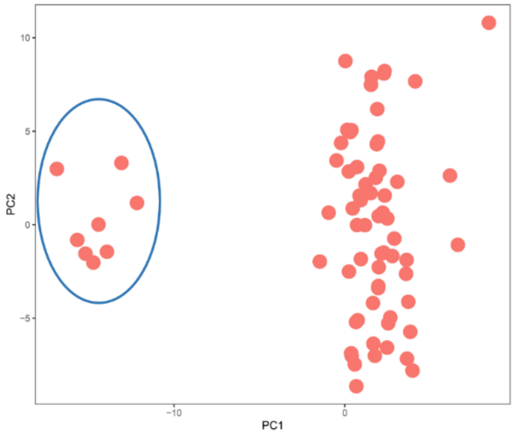


**Supplementary Figure 11. PCA plot of single cell level expression values.** PCA plot of expression values shows the separation of 8 normal oligodendrocytes from other tumor cells. Cells within blue circle corresponds to normal cells.

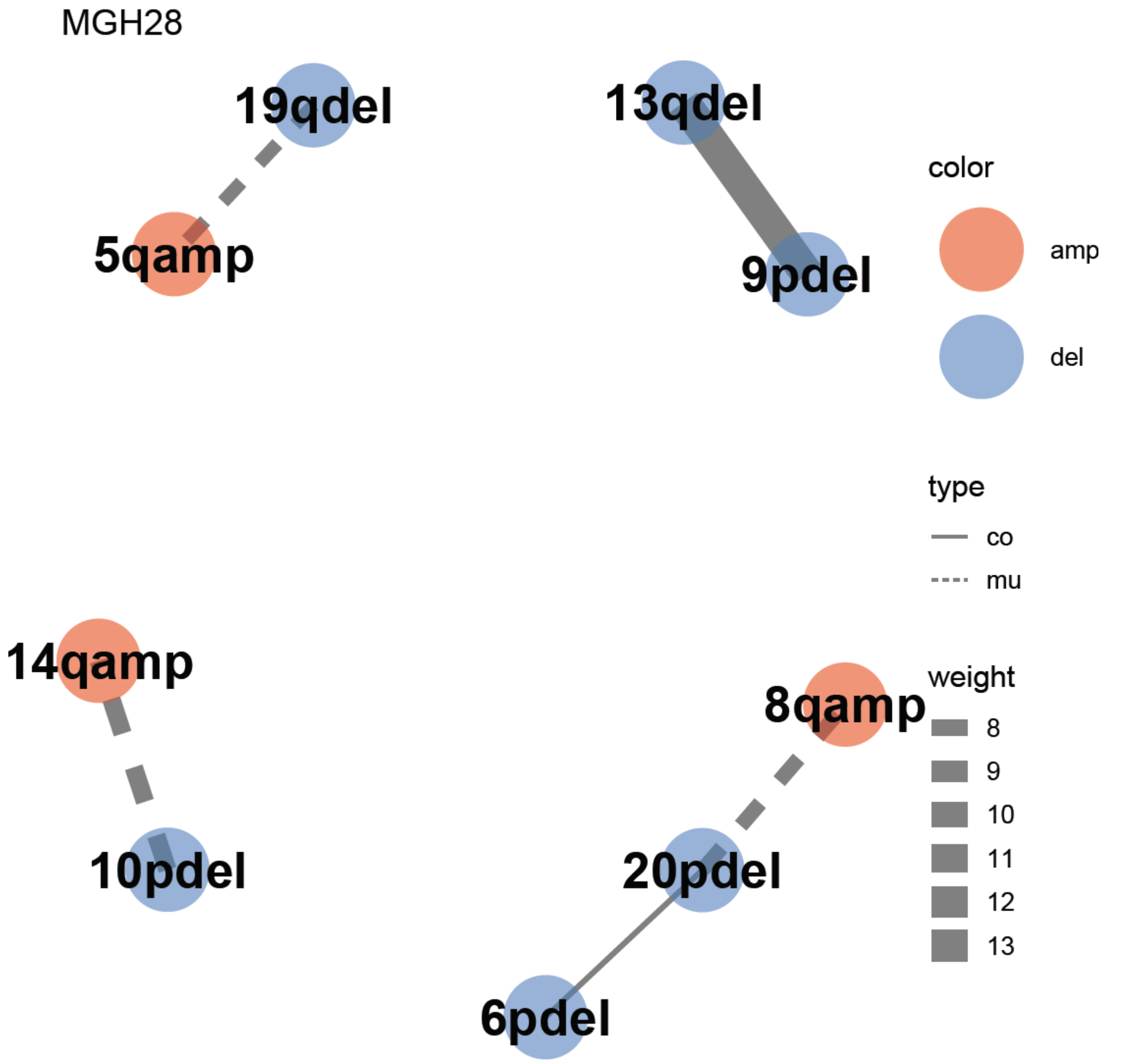


**Supplementary Figure 12. Mutual exclusive events in single-cell GBM data.** CaSpER detects mutually exclusive 8q:20p, 5q:19p event pairs for patient MGH28**.**

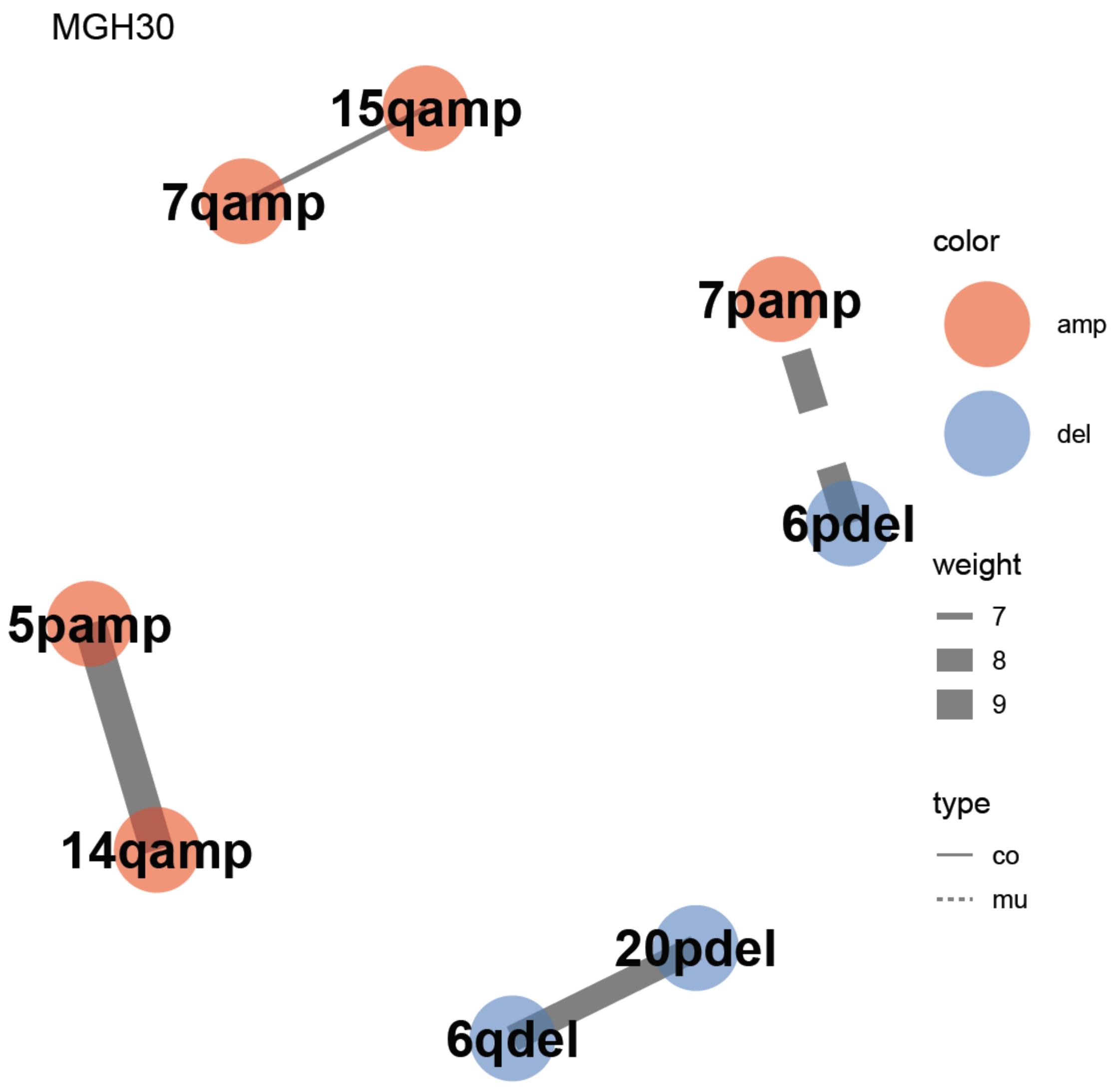


457 **Supplementary Figure 13. Mutual exclusive events in single-cell GBM data.** CaSpER detects

458 novel mutually exclusive 6p:7p event pairs for patient MGH30**.**

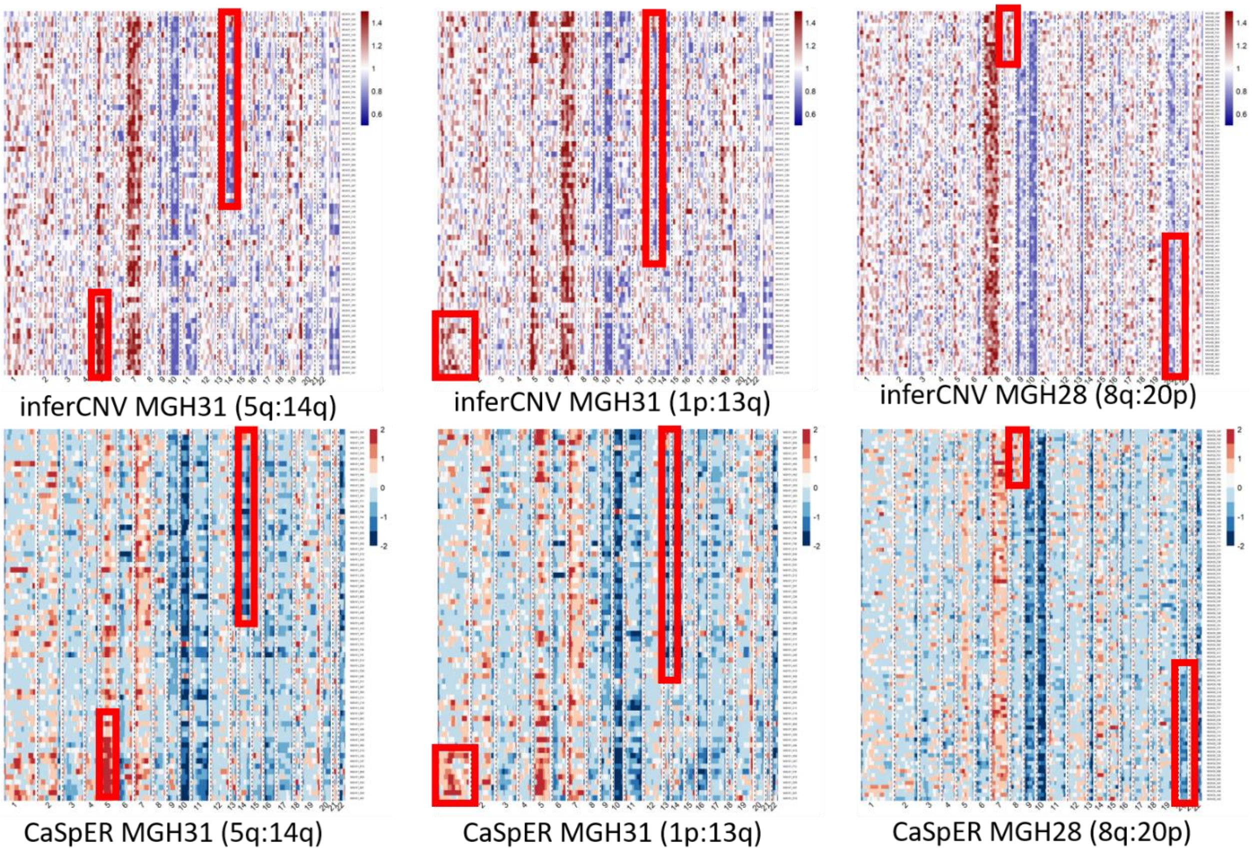


**Supplementary Figure 14**. **Heatmap plots of the expression signal generated by CaSpER and inferCNV.** Rows correspond to cells whereas columns correspond to genes ordered by chromosomal locations. Rows are ordered by the significant mutually exclusive event pairs identified by CaSpER.

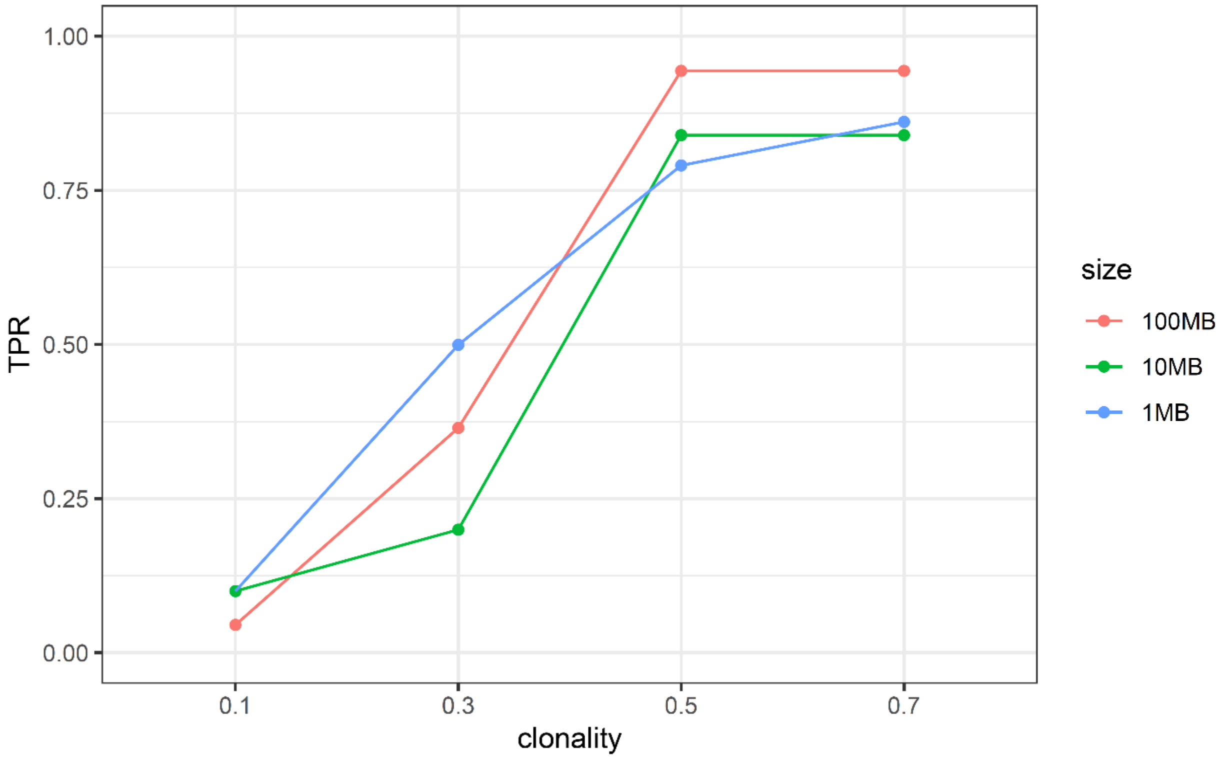


**Supplementary Figure 15. Plot of the sensitivity of the detected events with respect to the clonality.** Performance of CaSpER as a function of clonality. X-axis corresponds to varying levels of clonality; 10%, 30%, 50% and 70%. Y-axis corresponds to TPR value.

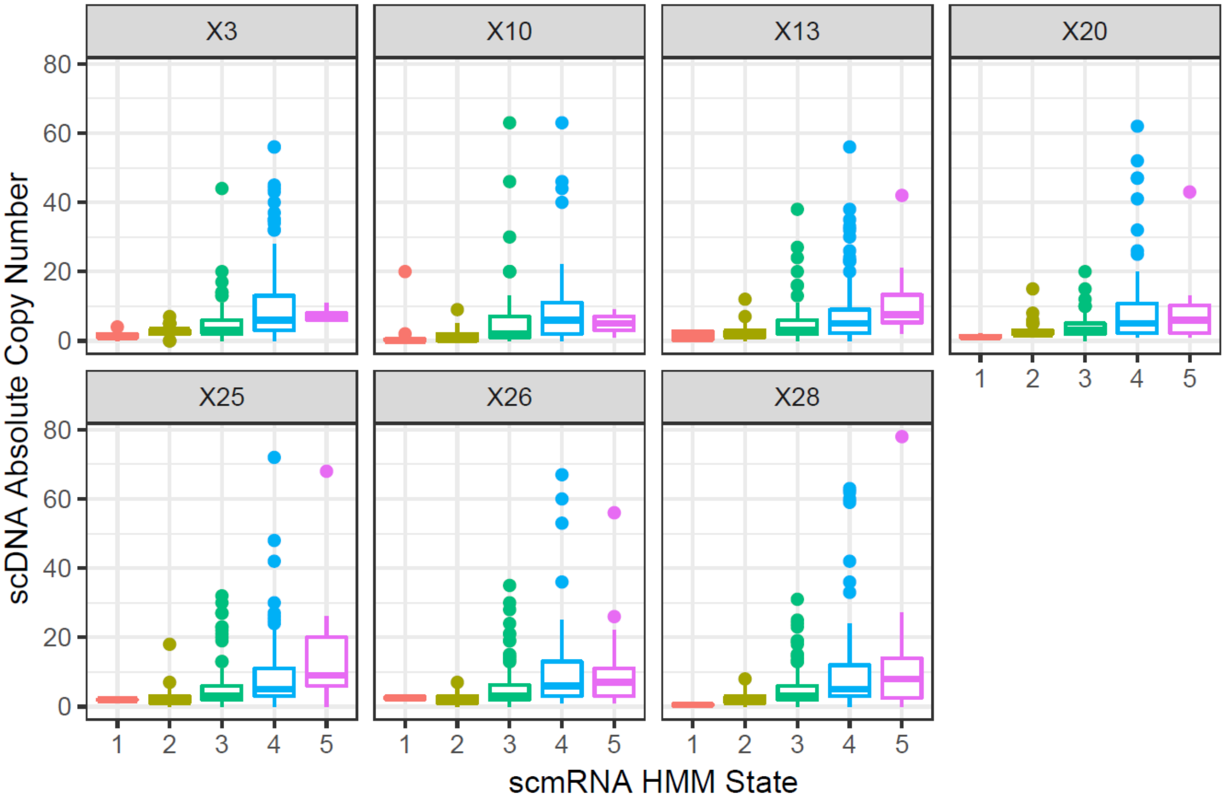


**Supplementary Figure 16**. **Accuracy of CNV events detected from DR-Seq study.** Figure shows, for each cell (X3, X10, X13, X20, X25, X26, X28; n=7) probed by the DR-Seq study, the distribution of the absolute copy number assigned to the genes (y-axis) vs the HMM state (x-axis). Box plots indicate the distribution of absolute copy number assigned to each gene by DR-Seq study, for the corresponding copy number state.

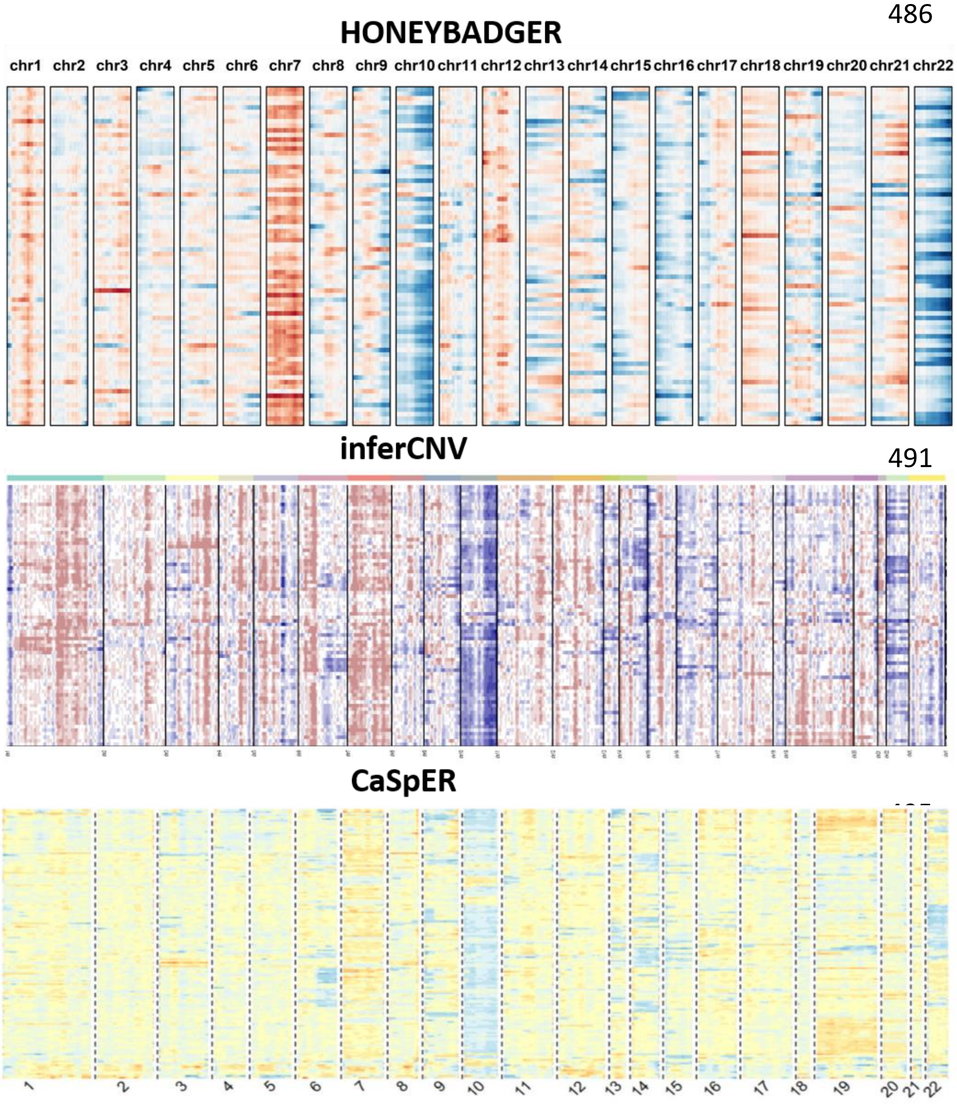


**Supplementary Figure 17. Expression plots of HoneyBADGER, inferCNV and CaSpER for TCGA-GBM data.**

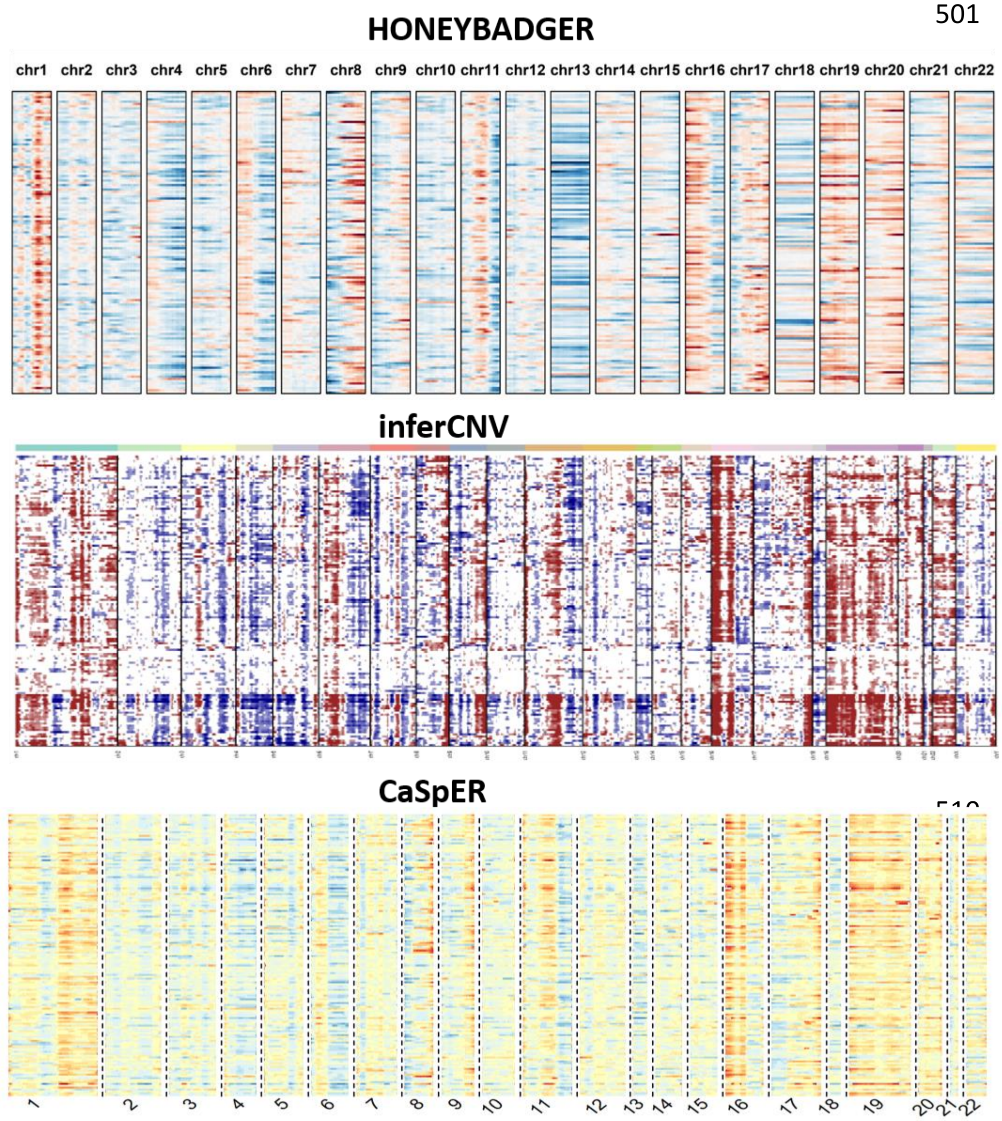


**Supplementary Figure 18.** Expression plot of HoneyBADGER, inferCNV and CaSpER for TCGA-BRCA data.

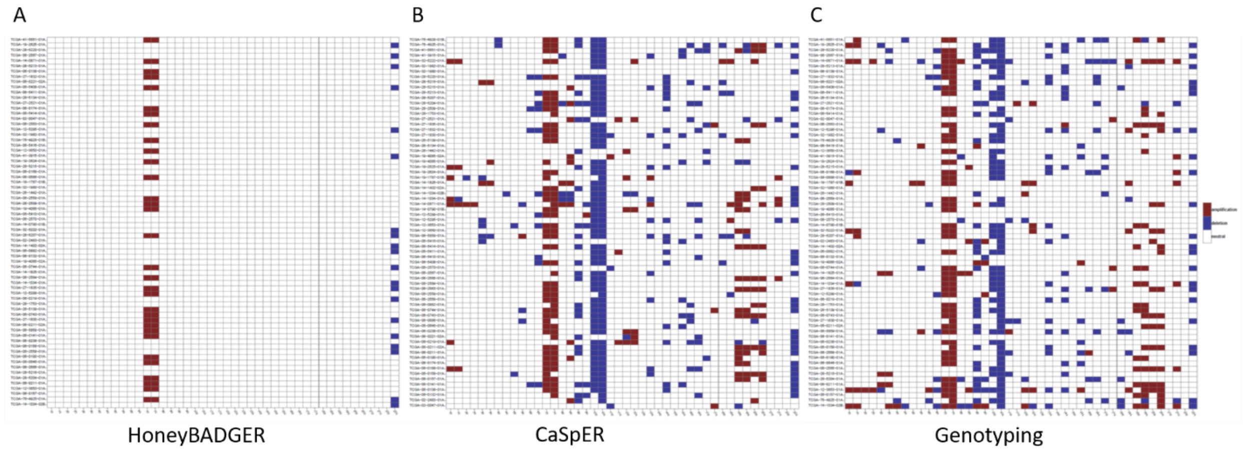


**Supplementary Figure 19.** Large scale event summary plots of HoneyBADGER (A), CaSpER (B) and Genotyping (C) for TCGA-GBM data. Rows correspond to samples whereas columns correspond to chromosome arms.

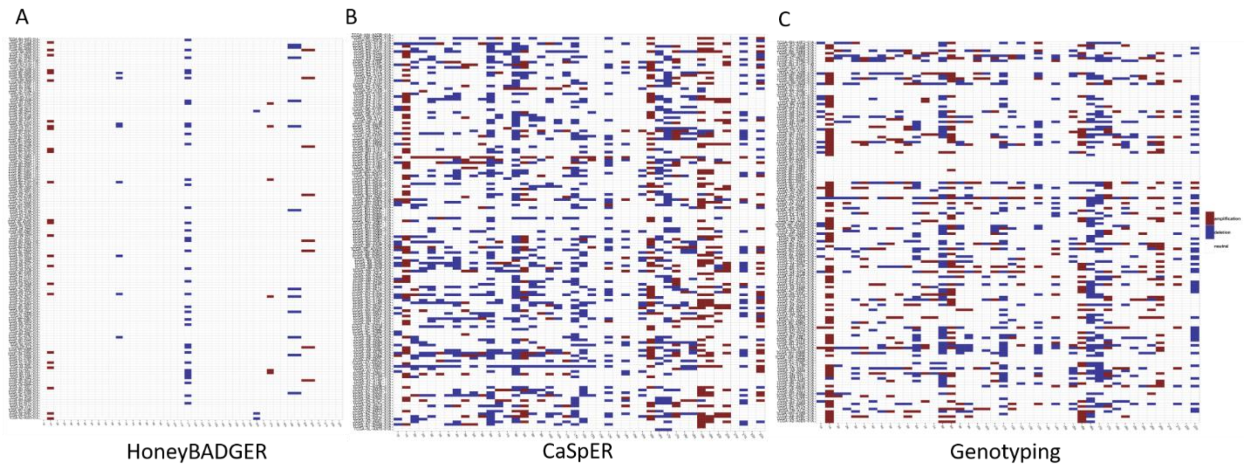


**Supplementary Figure 20.** Large scale event summary plots of HoneyBADGER (A), CaSpER (B) and Genotyping (C) for TCGA-BRCA data. Rows correspond to samples whereas columns correspond to chromosome arms.

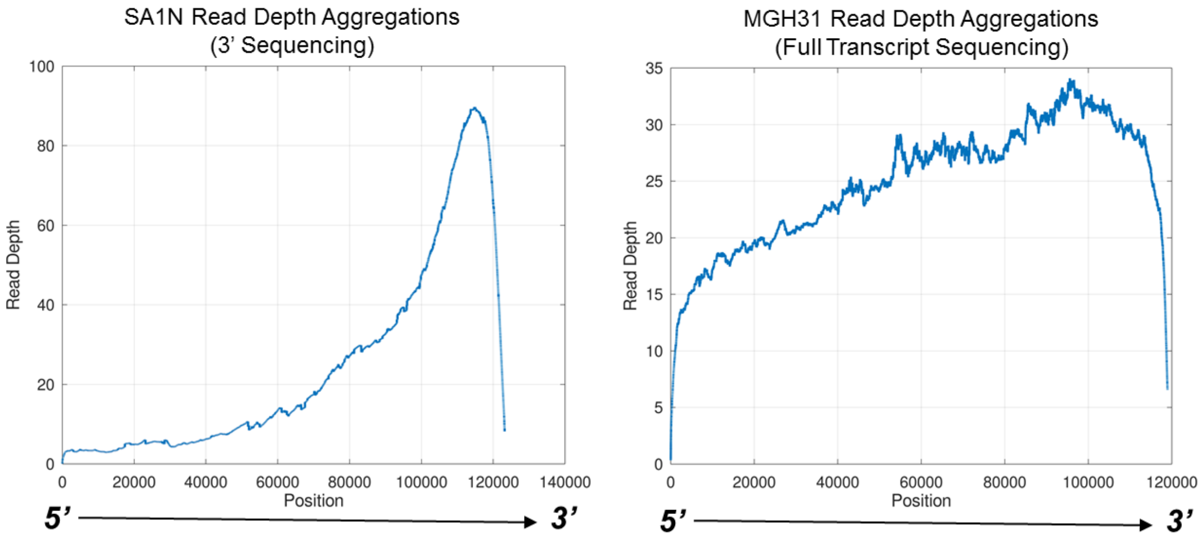


**Supplementary Figure 21. Comparison of read depth on exon-concatenated RNA-seq profiles.** Left plot shows the aggregation of 3' sequencing read depth. Right plot shows the aggregation of read depth on full transcript sequencing over the exon-concatenated genes. X-axis shows the normalized position on the concatenated exons. Y-axis shows the read depth. X-axis is oriented from 5' to 3'.

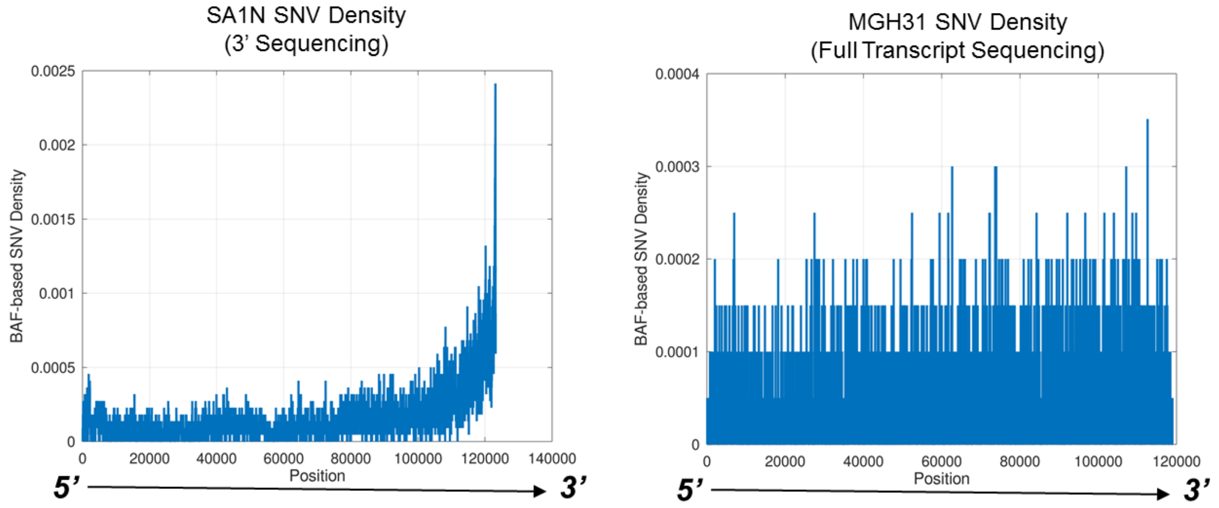


**Supplementary Figure 22. Comparison of SNV density on exon-concatenated genes as extracted by BAFExtract.** Left plot shows the SNV density from the 3' sequenced SA1N sample. Right plot shows the SNV density from the full transcript sequenced MGH31 sample over the exon-concatenated genes. X-axis shows the normalized position on the concatenated exons. Y-axis shows the read depth. X-axis is oriented from 5' to 3'.

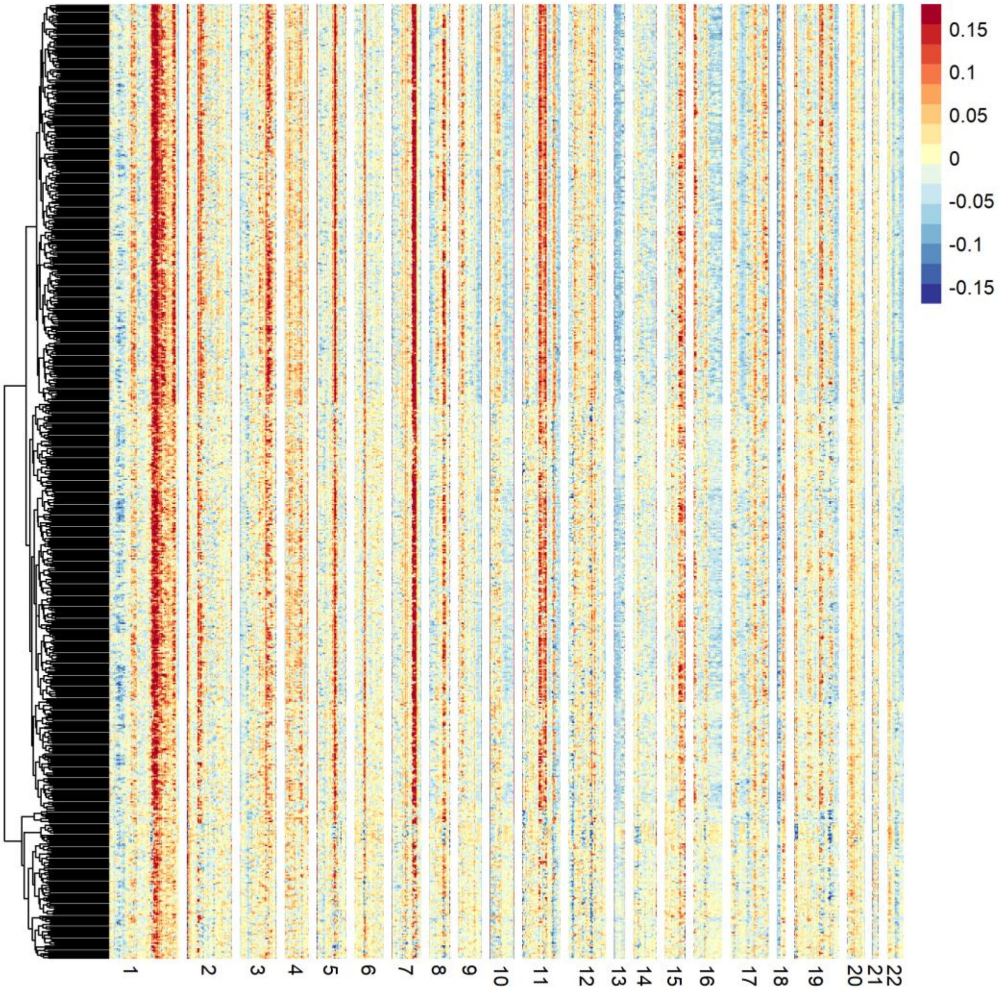


**Supplementary Figure 23. Heatmap of smoothed median filtered 10X RNA-Seq data.**

Whole chromosome chromosome 13, 16 and 22 deletion events can be seen from the heatmap.

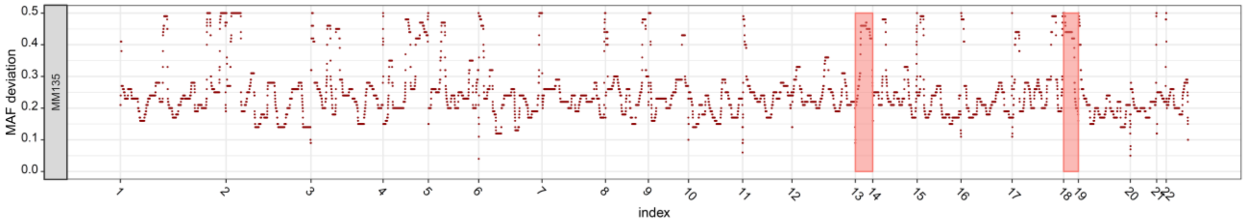


**Supplementary Figure 24. BAF shift plot for sample MM135.** Whole chromosome BAF shifts for chr 13 and 18 is highlighed.

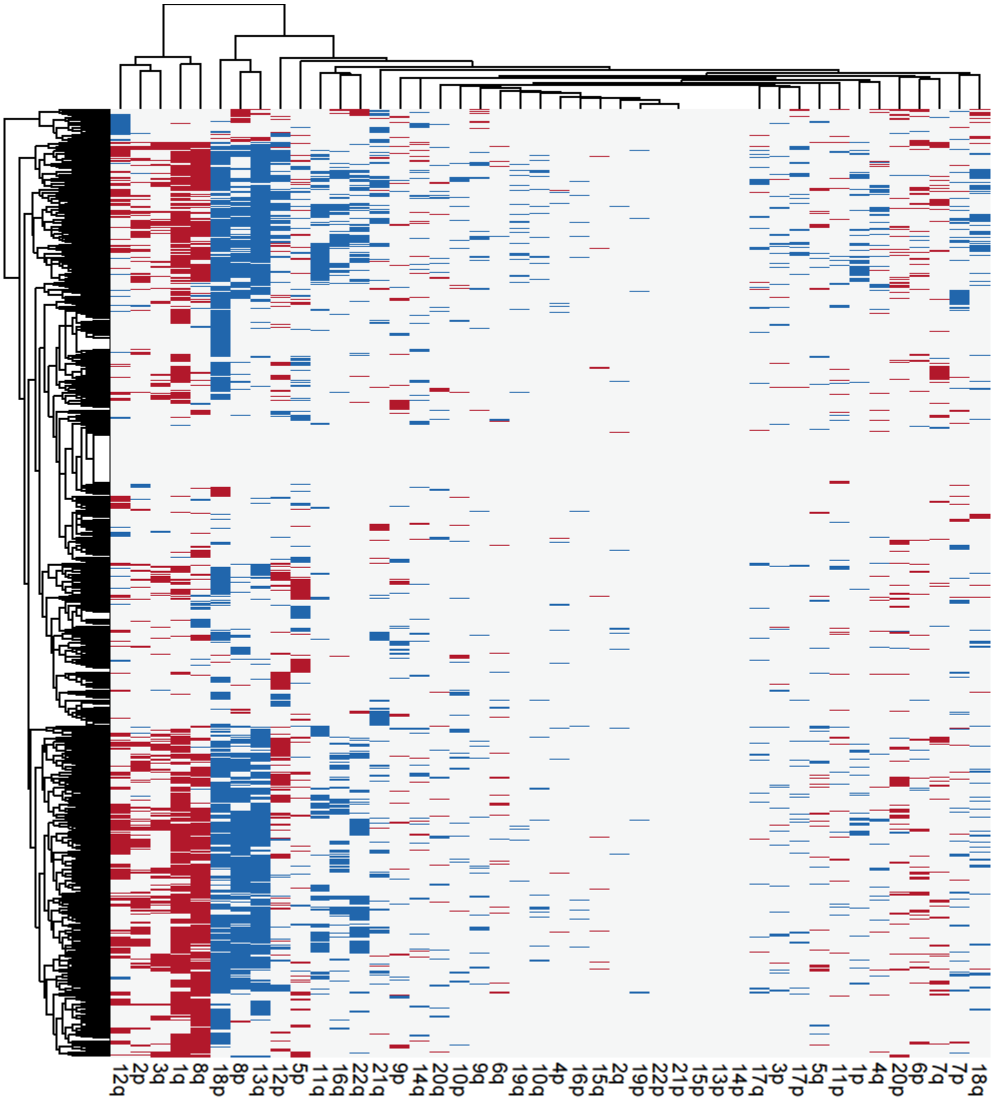


**Supplementary Figure 25. Large scale CNV event summary for MM135 data.**

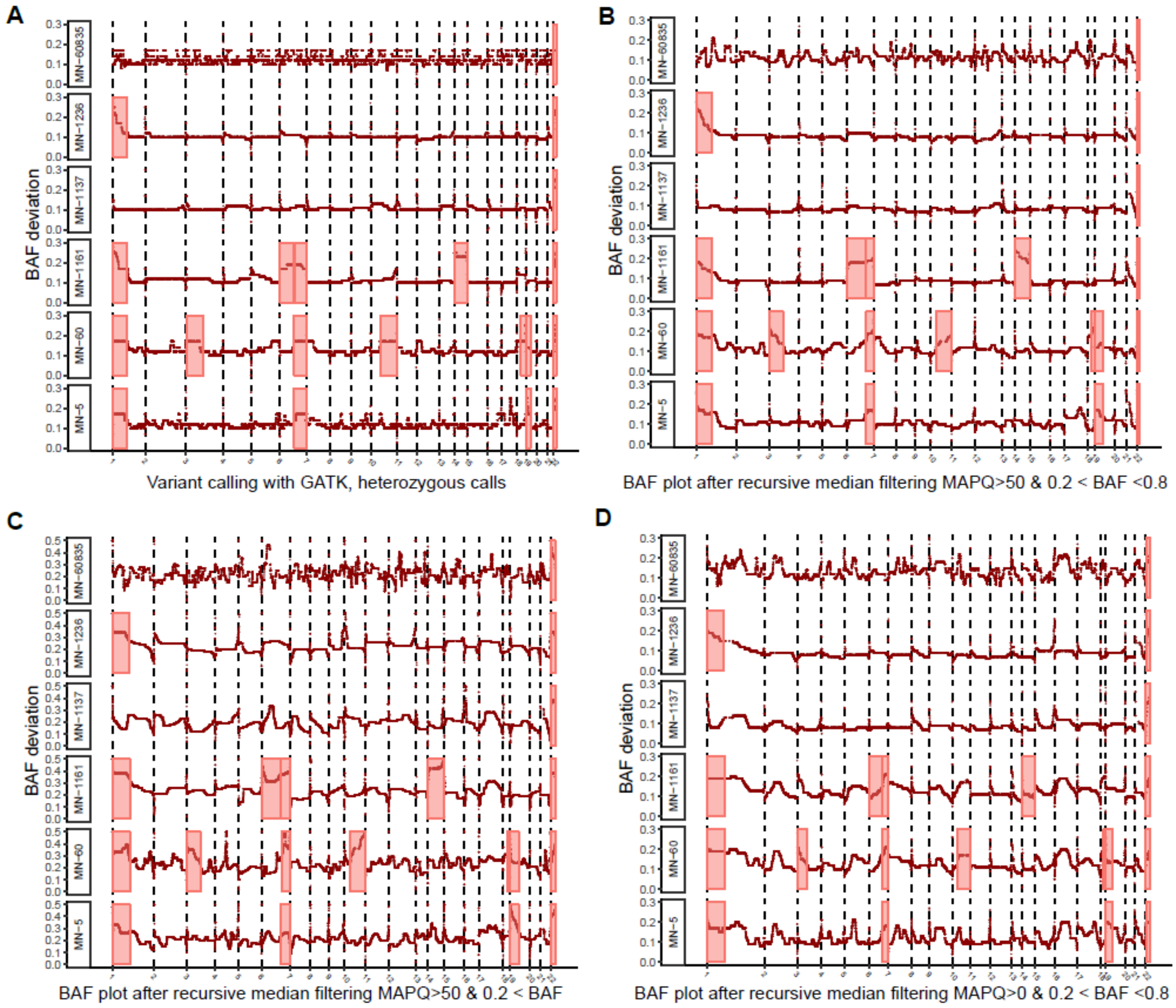


**Supplementary Figure 26. A.** BAF signal generated using GATK variant calling best practices. **B.** BAF signal generated after filtering reads with less than 50 mapping quality and only BAF values more than 0.2 and less than 0.8 are considered. **C.** BAF signal generated after filtering reads with less than 50 mapping quality and only BAF values more than 0.2 are considered. **D.** BAF signal generated using only BAF values that are more than 0.2 and less than 0.8.

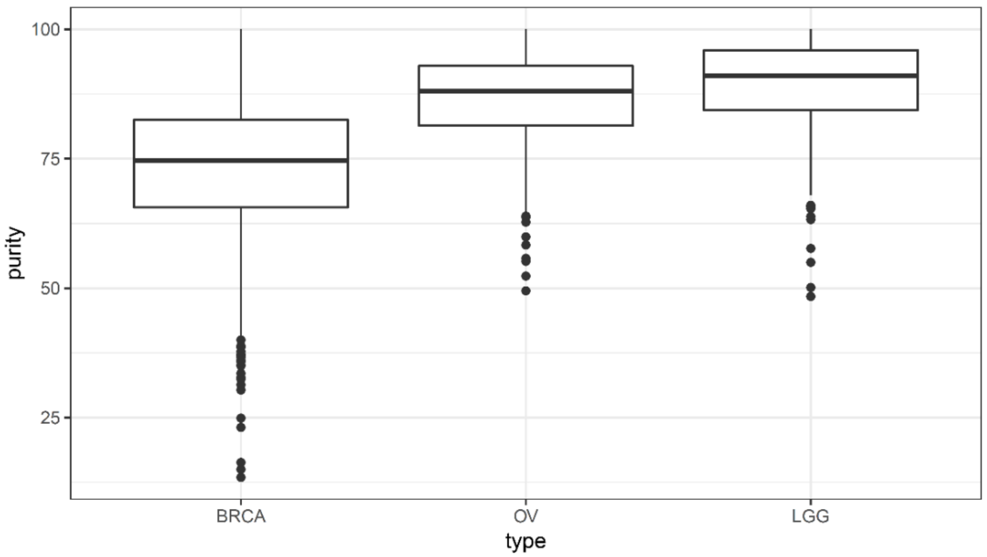


**Supplementary Figure 27.** Boxplot of tumor purity percentages across different cancer types in TCGA cohort. Breast cancer (BRCA), Ovarian cancer: OV, Low grade glioma: LGG. Lines depict the median values; boxes plot 25th to 75th percentiles, whereas separately plotted dots show the outliers.

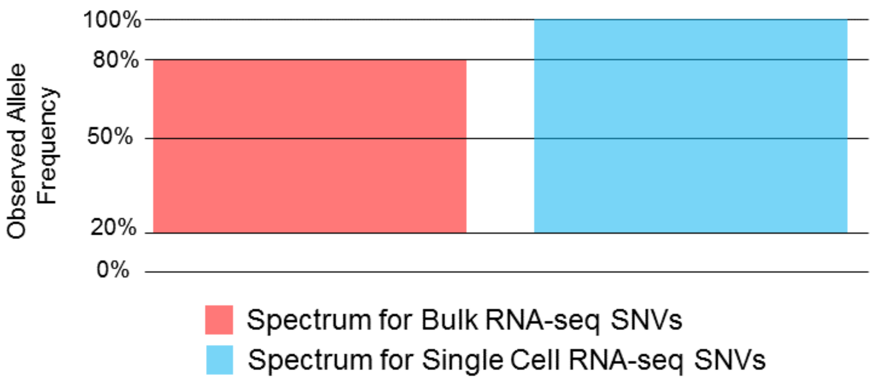


**Supplementary Figure 28. The illustration of the observed allele frequency ranges (or spectrums) that are used to select the SNVs for computing the BAF shift signal.** For bulk RNA-seq, the SNVs whose observed allele frequencies are in 20%-80% range (Illustrated with red box) are selected for computing the BAF shift signal. For single cell RNA-seq, the SNVs whose observed allele frequencies are in the range 20%-100% (Illustrated with blue box) are used for computing the BAF shift signal.

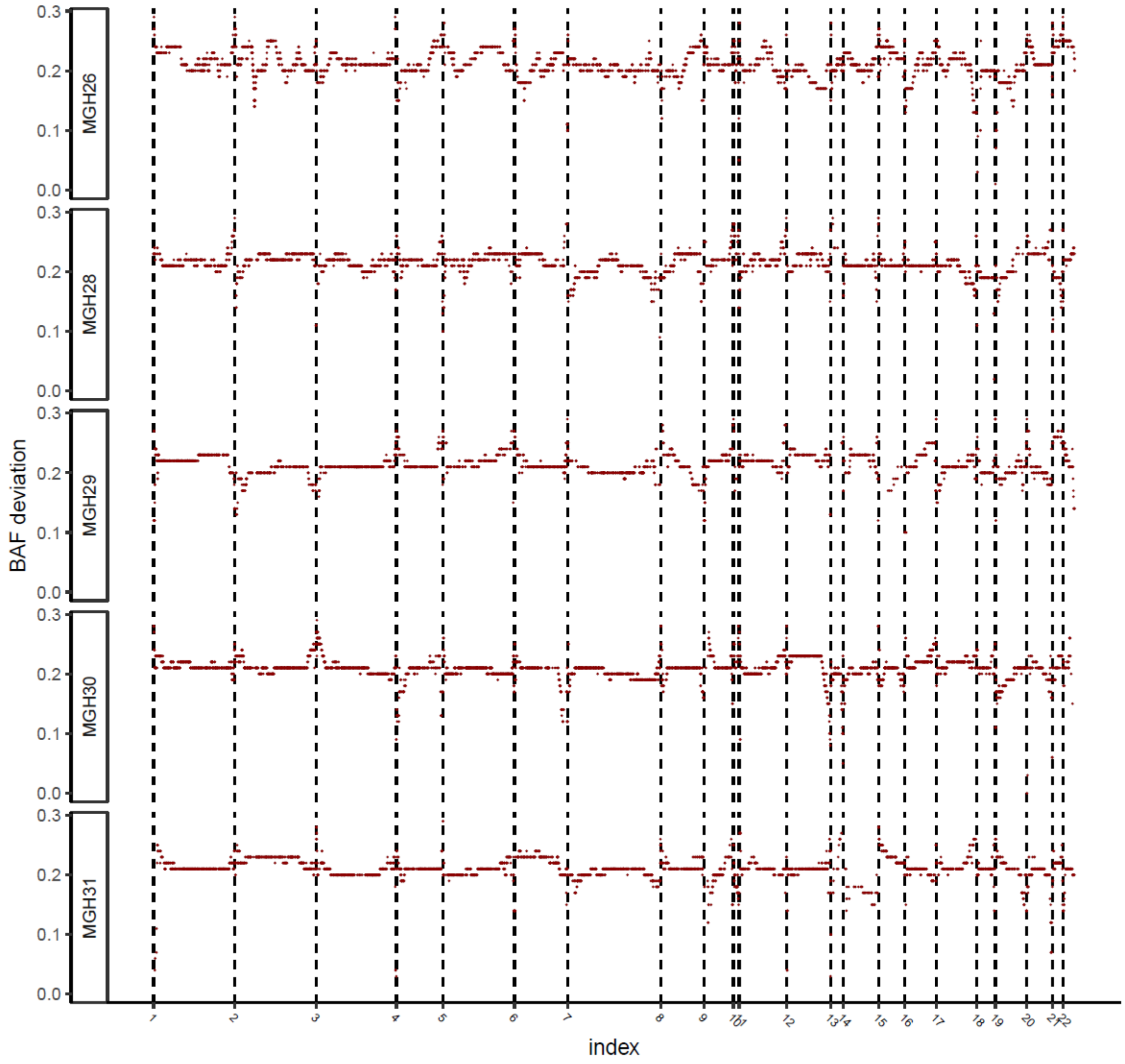


**Supplementary Figure 29. BAF signal generated from single-cell GBM RNA-Seq.** BAF signal generated from single-cell GBM RNA-Seq data using only BAF values that are more than 0.2 and less than 0.8. BAFshifts can not be detected using these thresholds.

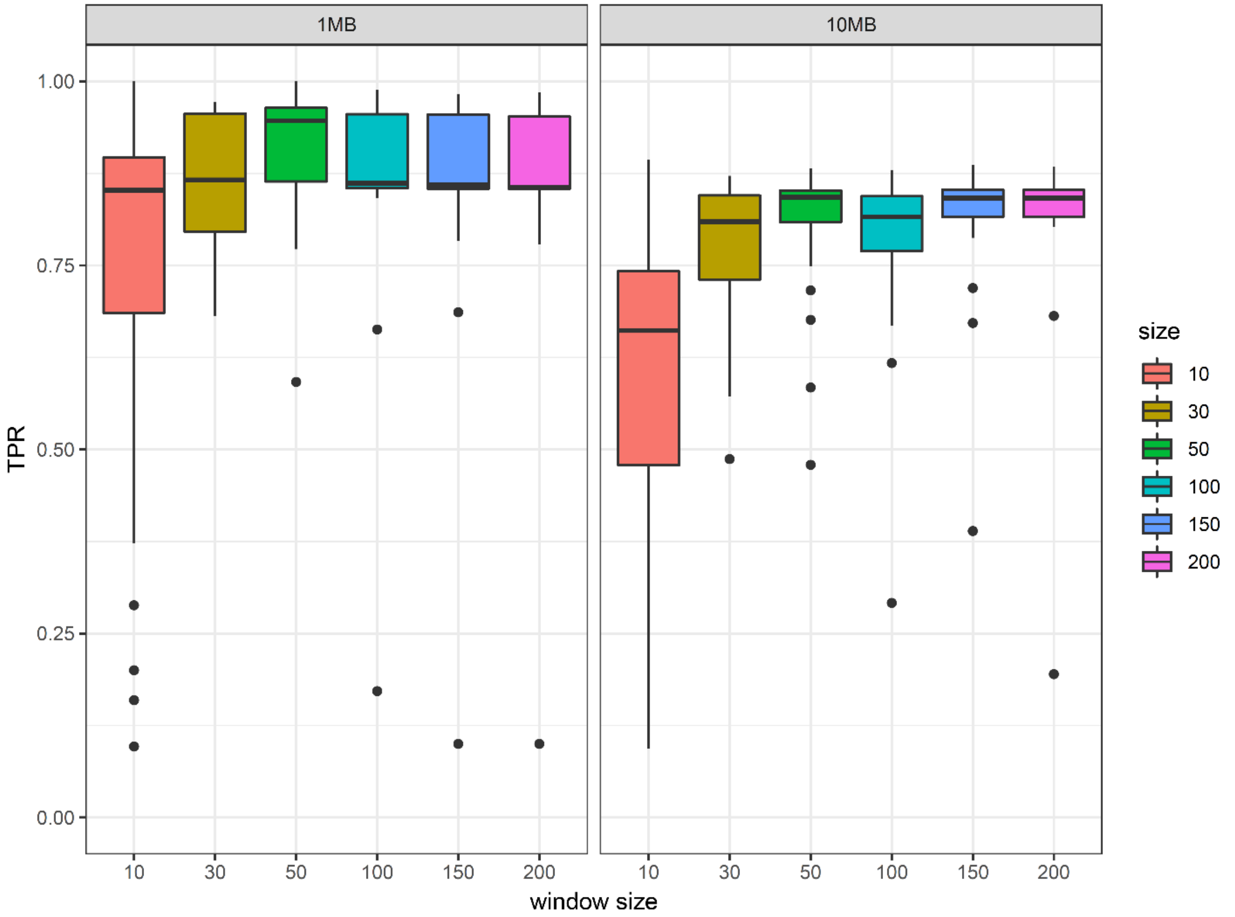


**Supplementary Figure 30. TPR values of the CNV calls made by CaSpER using the GBM single cell RNA-seq data in which we simulated introduction of deletions at 1MB and 10MB.** For small smoothing window sizes, segments show relatively low concordance with the ground truth. As the window size increases, the concordance increases and saturates around 80% at the around window length of 50 for both event lengths. Lines depict the median values; boxes plot 25th to 75th percentiles, whereas separately plotted dots show the outliers.

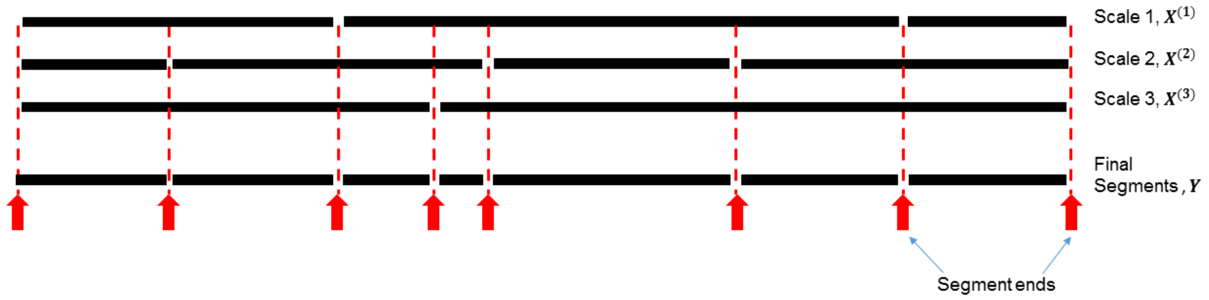


**Supplementary Figure 31. A toy example for detection of final consistent segments.**

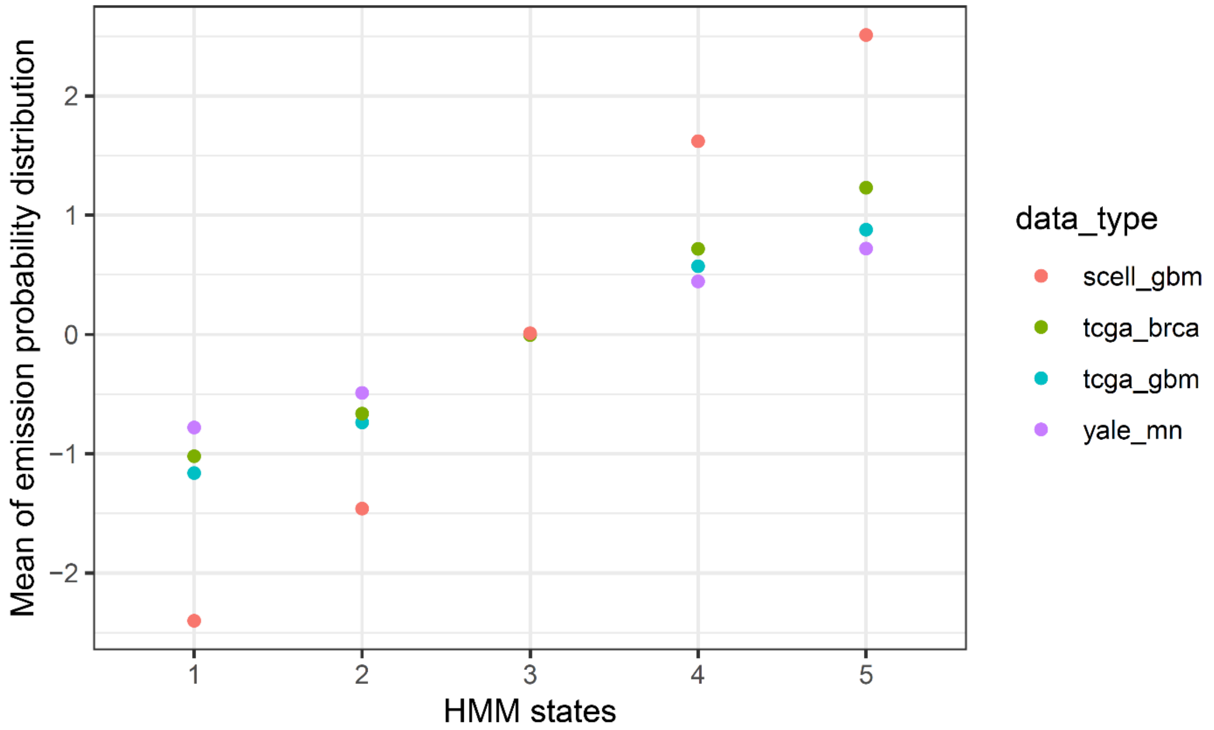


**Supplementary Figure 32. Mean values of the normal distributions corresponding to 5 copy number states of different datasets**. Mean values of the normal distributions corresponding to 5 copy number states (homozygous deletion, heterozygous deletion, neutral, amplification, high-level amplification) that are derived from the datasets; i.e. single cell GBM, bulk TCGA GBM, bulk TCGA-BRCA and bulk meningioma datasets. X-axis shows the HMM states and Y-axis shows the mean of the Gaussian emission probability for each state. Single cell RNA-seq data are assigned lower expression distributions at the deletion states (states 1, 2) and higher expression distributions at the amplification states (states 4, 5).

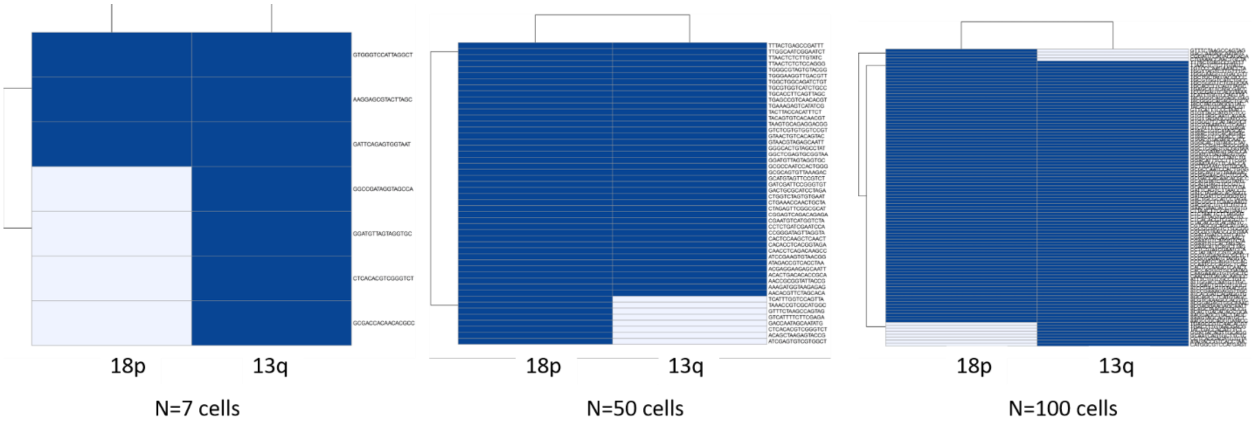


**Supplementary Figure 33. Concordance of CNV calls with increasing number of cells in MM135 dataset.** Large scale CNV calls for chromosome 18 and 13 for randomly selected n=7, n=50 and n=100 cells that are known to harbor these deletion events. Blue cells correspond to the deletion event whereas light blue cells correspond to neutral events. Rows correspond to single cells.

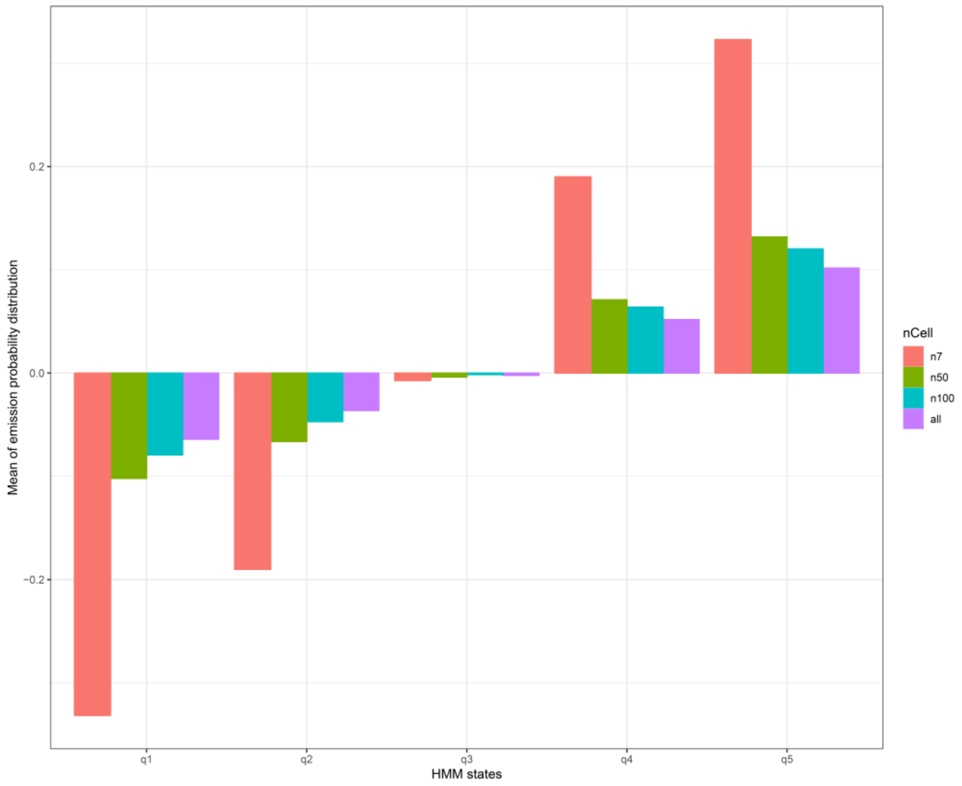


**Supplementary Figure 34. Mean values of the normal distributions corresponding to 5 copy number states in MM135 dataset.** Mean values of the normal distributions corresponding to 5 copy number states (q1:homozygous deletion, q2:heterozygous deletion, q3:neutral, q4:amplification, q5:high-level amplification) that are calculated from randomly selected n=10, n=50, n=100 and all cells in MM135 dataset.

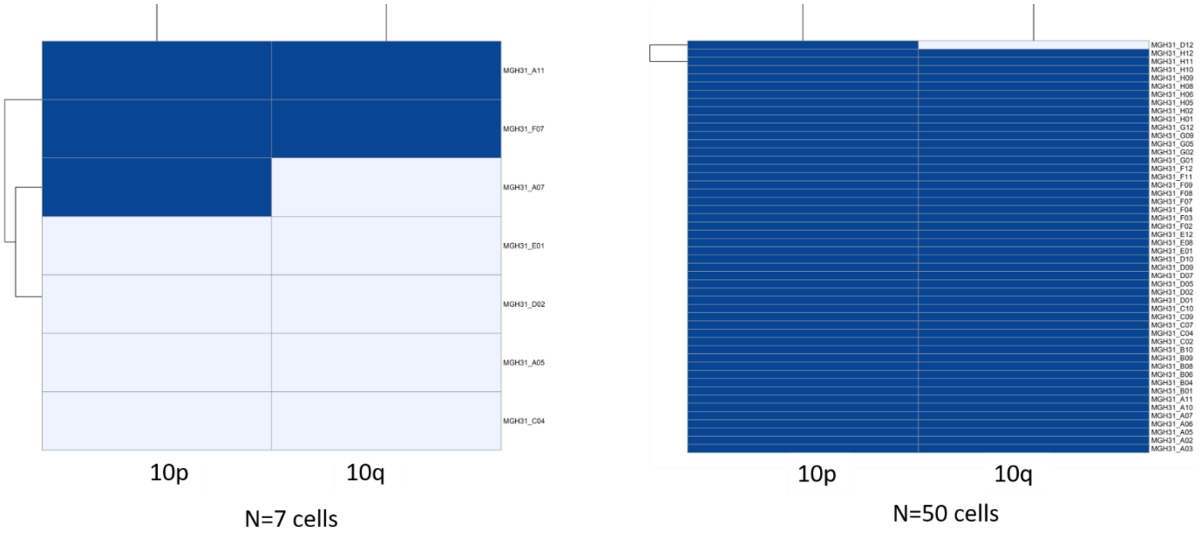


**Supplementary Figure 35. Concordance of CNV calls with increasing number of cells in MGH31 dataset.** Large scale CNV calls for chromosome 10p and 10q for randomly selected n=7 and n=50 cells that are known to harbor these deletion events. Blue cells correspond to deletion event whereas light blue cells correspond to neutral events. Rows correspond to single cells.

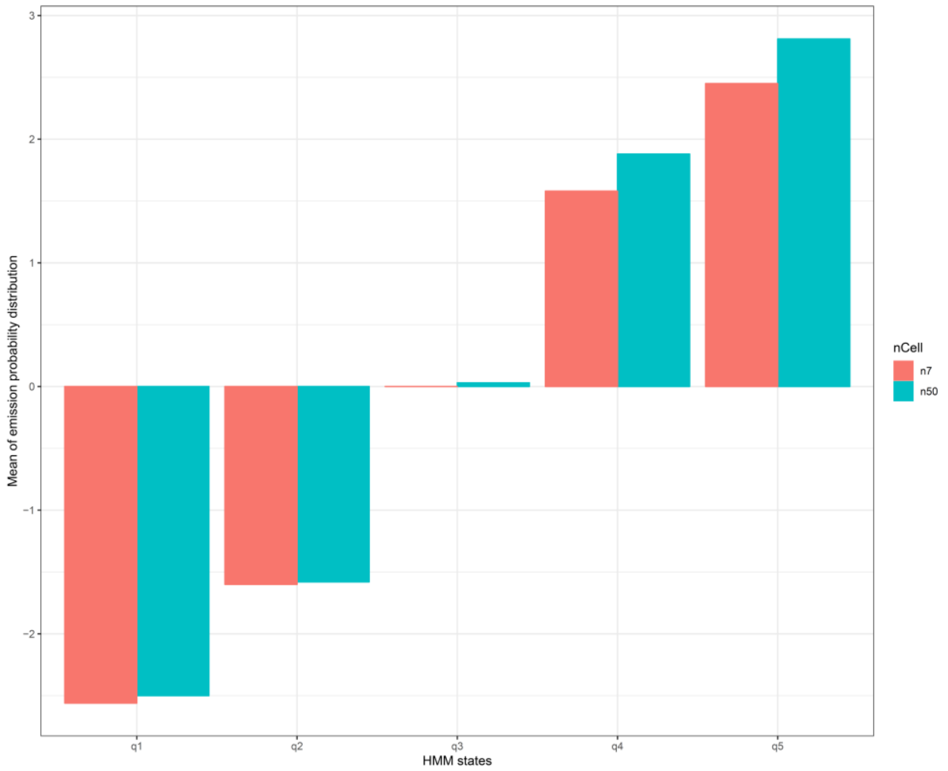


**Supplementary Figure 36. Mean values of the normal distributions corresponding to 5 copy number states in MGH31 dataset.** Mean values of the normal distributions corresponding to 5 copy number states (q1:homozygous deletion, q2:heterozygous deletion, q3:neutral, q4:amplification, q5:high-level amplification) that are calculated from randomly selected n=7 and n=50 in MGH31 dataset.

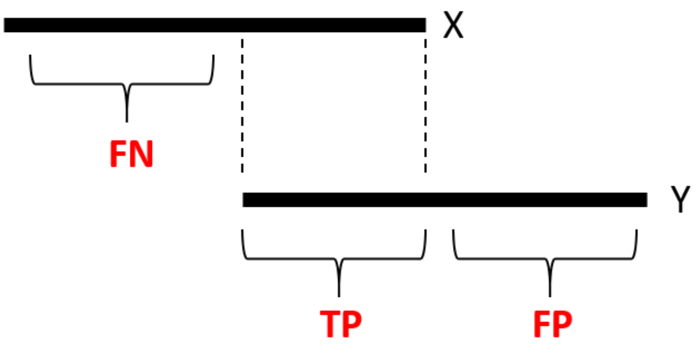


**Supplementary Figure 37. Illustration of the false negative, false positive, and true positive portions of the segments.**

## Supplementary Tables

| | | Large Scale Level | | Gene Level | |
|---|---|---|---|---|---|
| | | HoneyBADGER | CaSpER | HoneyBADGER | CaSpER |
| TPR | Deletion | 6% | 85% | 5.9% | 77.6% |
| | Amplification | 25% | 71% | 22% | 57.5% |
| FPR | Deletion | 0.07% | 3% | 0.27% | 3.5% |
| | Amplification | 0.14% | 1.5% | 0.23% | 2% |

**Supplementary Table 1. TPR and FPR values for bulk RNA-Seq TCGA-GBM**

| | | Large Scale Level | | Gene Level | |
|---|---|---|---|---|---|
| | | HoneyBADGER | CaSpER | HoneyBADGER | CaSpER |
| TPR | Deletion | 6.4% | 79% | 3.2% | 62.8% |
| | Amplification | 8% | 60.3% | 10.5% | 56.6% |
| FPR | Deletion | 0.46% | 7.7% | 0.75% | 6% |
| | Amplification | 0.1% | 3.8% | 0.09% | 7% |

**Supplementary Table 2. TPR and FPR values for bulk RNA-Seq TCGA-BRCA**

| | | True events | |
|---|---|---|---|
| | | Alteration event | No alteration event |
| Predicted events | Predicted alteration event | TP | FP |
| | Predicted no alteration event | FN | TN |

**Supplementary Table 3. Definition of true positive and false positive rate values**
